# One-Pot in Situ Hydrothermal Growth of BiVO_4_/Ag/rGO Hybrid Architectures for Solar Water Splitting and Environmental Remediation

**DOI:** 10.1038/s41598-017-08912-z

**Published:** 2017-08-21

**Authors:** Santosh S. Patil, Mukund G. Mali, Mostafa Afifi Hassan, Deepak R. Patil, Sanjay S. Kolekar, Sang-Wan Ryu

**Affiliations:** 1 Department of Physics, Chonnam National University, Gwangju, 61186 Republic of Korea; 2 School of Chemical Sciences, Solapur University, Solapur, MS India; 3 Centre for Materials for Electronics Technology (C-MET), Department of Electronics and Information Technology (DeitY), Govt. of India, Pune, India; 4 Analytical Chemistry and Material Science Laboratory, Department of Chemistry, Shivaji University, Kolhapur, India

## Abstract

BiVO_4_ is ubiquitously known for its potential use as photoanode for PEC-WS due to its well-suited band structure; nevertheless, it suffers from the major drawback of a slow electron hole separation and transportation. We have demonstrated the one-pot synthesis of BiVO_4_/Ag/rGO hybrid photoanodes on a fluorine-doped tin oxide (FTO)-coated glass substrate using a facile and cost-effective hydrothermal method. The structural, morphological, and optical properties were extensively examined, confirming the formation of hybrid heterostructures. Ternary BiVO_4_/Ag/rGO hybrid photoanode electrode showed enhanced PEC performance with photocurrent densities (*J*_*ph*_) of ~2.25 and 5 mA/cm^2^ for the water and sulfate oxidation, respectively. In addition, the BiVO_4_/Ag/rGO hybrid photoanode can convert up to 3.5% of the illuminating light into photocurrent, and exhibits a 0.9% solar-to-hydrogen conversion efficiency. Similarly, the photocatalytic methylene blue (MB) degradation afforded the highest degradation rate constant value (*k* = 1.03 × 10^−2^ min^−1^) for the BiVO_4_/Ag/rGO hybrid sample. It is noteworthy that the PEC/photocatalytic performance of BiVO_4_/Ag/rGO hybrid architectures is markedly more significant than that of the pristine BiVO_4_ sample. The enhanced PEC/photocatalytic performance of the synthesized BiVO_4_/Ag/rGO hybrid sample can be attributed to the combined effects of strong visible light absorption, improved charge separation-transportation and excellent surface properties.

## Introduction

The rapid fading of fossil fuel resources, elevated level of greenhouse gas emission, and growing water pollution due to the vast industrialization and population growth have become a serious threat to the environmental and economic stability of the world^1–3^. By recognizing potential hazards, researchers worldwide have devoted their principal efforts to prevent these overwhelming issues by developing highly advanced environmental technologies for a sustainable future. Besides, industrialists and even householders are seriously looking for alternative ecofriendly renewable energy resources. Remarkably, the beautiful planet earth is gifted with two of the most abundant and limitless energy resources, namely sun and water, thus offering a stockpile of renewable energy. However, a breakthrough technology that could effectively harvest and implement these resources still needs to be implemented. The direct solar water splitting (WS) using photoelectrochemical (PEC) cells represents one of the potential and exciting ways to convert the solar energy into chemical energy. This process can split H_2_O into molecular hydrogen (H_2_) and oxygen (O_2_) under the influence of a photocatalyst and sunlight energy. The H_2_ produced by the PEC-WS process is 100% clean over its generation cycles and can be stored for future use and even used directly wherever the fossil fuels are currently used. The last three decades have witnessed the importance of this hotspot research topic with rapid progresses in this field; however, the efficiency with which H_2_ is produced is still too low to meet the industrial requirements. The major bottleneck to achieve a high efficiency of PEC-WS lies in the lack of highly efficient, robust, and inexpensive photoelectrode materials. The main parameters accounting for the PEC-WS cell performance are (i) significant absorption of the solar spectrum by photocatalytic materials, (ii) efficient separation of the photoexcited charge carriers and their transport, (iii) reusability/stability of the photocatalytic materials, and (iv) surface chemical reactions^4, 5^. Among these, the most influential factor is represented by the surface chemical reactions. This is due to the fact that the photoexcited charge carriers (e^−^/h^+^) in the photochemical reactions should be captured very quickly by the targeted molecules. Otherwise, if they recombine, no reaction would occur and this is very critical in the design of an efficient photoelectrode material for PEC-WS cells. In fact, the rational design of highly efficient photoanodes is still being actively sought.

Monoclinic N-type bismuth vanadate (*m*-BiVO_4_) is one of the most promising photoanode materials for PEC-WS cells due to its low cost, well-suited band structure^6^, high optical absorption coefficient (10^4^–10^5^ cm^−1^ at *hν* = 2.5–3.5 eV)^7^, and favorable flat band potential (<200 mV) positive to the H_2_ evolution reaction (HER)^8^, which permits the O_2_ evolution at a lower bias than several other metal oxides^8, 9^. Moreover, BiVO_4_ can translate the maximum theoretical photocurrent of ~7.5 mA/cm^2^ at 1.23 V *vs*. RHE at 1.5 AM light (100 mW/cm^2^). However, BiVO_4_ photoanodes often suffer from a poor charge carrier transport together with a bulk carrier mobility of 0.05–0.2 cm^2^ V^−1^ s^−1^ at room temperature and hopping activation energy of ~0.3 eV^8, 10^. The photoactivity (solar to hydrogen conversion efficiency) of BiVO_4_ acquired to date is far too low (<1%) with a low photocurrent density of <1 mA/cm^2^ at 1.23 V *vs*. RHE, because most of the photogenerated charge carriers (e^−^/h^+^) recombine in the bulk of BiVO_4_ before the reaction^11^. The foremost reason for the fast recombination of e^−^/h^+^ is the short diffusion length of the photogenerated charge carriers in the bulk of BiVO_4_^11, 12^. To circumvent these limitations of BiVO_4_, several strategies such as the design of nanostructures, metal doping (Mo, W)^10, 13^, and heterostructure construction with other semiconductors^14, 15^ have been employed. Among these, the heterostructure construction strategy through coupling of BiVO_4_ with other appropriate semiconductor materials (e.g. BiVO_4_/ZnFe_2_O_4_^16^, WO_3_/BiVO_4_^11^, TiO_2_/BiVO_4_^17^, BiVO_4_/SnO_2_/WO_3_^18^, and BiVO_4_/FeOOH^19^) has led to a significant improvement of the PEC performance. Previous studies on heterostructure photocatalysts revealed their several advantages over single-phase photocatalysts such as the improvement of the overall light harvesting ability, enhancement of the charge carrier mobility, and better structural stability, which resulted in a higher efficiency of the corresponding PEC-WS devices. In particular, when we consider the electronic structure of BiVO_4_, the valence band (VB) of BiVO_4_ is 1 V more positive than the onset of the thermodynamic potential for water oxidation (1.23 V *vs*. RHE); similarly the conduction band (CB) is also located close to the RHE, thereby the heterostructure construction with other semiconductor and conductor materials is favored, smoothly enabling high PEC-WS performances at low bias potentials^20^.

In the past decade, many heterostructure photocatalysts have been developed by modification with carbonaceous materials^21^ or decoration with noble metal nanoparticles (NPs)^22, 23^. Among these, noble metal NPs/semiconductor heterostructures have attracted great attention because of their strong absorption in the visible light region due to the so-called localized surface plasmon resonance (LSPR) effect^14^. This LSPR effect helps to improve the optical-to-chemical energy conversion efficiency, interfacial charge transfer kinetics, and photostability. Moreover, plasmonic metal nanoparticles such as Ag, Au, Pt, etc., have the ability to confine the light in the vicinity of their surfaces facilitating the charge generation and separation, thereby improving the overall solar PEC-WS efficiency^24^. For example, Warren *et al*. have evaluated α-Fe_2_O_3_ for PEC-WS cells and found enhanced water splitting activity at photon frequencies related to the plasmonic resonance in Au^25^. A size dependent plasmonic effect of Au nanoparticles on BiVO_4_ photoanodes for water splitting has been demonstrated by Zhang and coworkers which resulted into 2.5 fold enhancement into photoactivity^26^. Similarly, the compatibility between Ag and BiVO_4_ for the construction of photoanode heterostructures was investigated in some recent reports. Ag is a cheaper metal compared to Pt and Au, and exhibits high electrical conductivity, strong surface plasmon resonance, and excellent energy conversion efficiency. Most recently, Fang *et al*. have demonstrated the synthesis of BiVO_4_/Ag heterostructures by a nanosphere lithography route combined with the pulsed current deposition method, which indeed led to a 4.3 time enhancement of the PEC-WS performance compared to pristine BiVO_4_^12^. However, one of the main limitations of this type of system is that the interaction between the photocatalyst and Ag NPs is relatively weak. Thus, the loose connections between the different components consequently impede the interfacial charge transfer. To overcome these issues, the design of ternary hybrid heterostructures prepared by wrapping BiVO_4_/Ag binary heterostructures with reduced graphene oxide (rGO) is necessary to endow the photocatalysts with a high surface-to-volume ratio and abundant reactive sites. Owing to its excellent surface and electronic properties, rGO could improve the PEC-WS efficiency when hybridized with BiVO_4_ by providing not only more surface active sites for charge transfer, but also assisting in establishing a superior electrical contact with the conducting FTO glass substrates. It is noteworthy that previously reported binary photoanodes based on BiVO_4_/Ag and BiVO_4_/rGO heterostructures were separately illustrated; however, their combined effect in ternary rGO wrapped BiVO_4_/Ag heterostructures has not been examined and reported so far for PEC-WS devices. Therefore, the synthesis of ternary BiVO_4_/Ag/rGO heterostructures and the fundamental understanding of their chemistry are desirable, as they may be helpful to evaluate the underlying photocatalytic reaction mechanisms and correlate the reaction parameters with the photocatalytic activity.

In this study, we report a simple chemical route to fabricate BiVO_4_/Ag/rGO hybrid photoanodes using a hydrothermal method. It is important to note that most of the noble metal/BiVO_4_ thin film photoanodes are fabricated by using physical synthesis methods that generally require high temperatures and expensive equipment, limiting their large-scale synthesis. Therefore, the hydrothermal synthesis of BiVO_4_/Ag/rGO hybrid architectures is very advantageous due to the low temperature, modest cost, large scalability, and relatively simple fabrication procedure. The physicochemical properties of the BiVO_4_/Ag/rGO hybrid samples were extensively studied using different characterization techniques and utilized in PEC-WS cells irradiated with an AM1.5 G light. The photocatalytic activities of the BiVO_4_/Ag/rGO hybrid architectures were also tested for methylene blue (MB) degradation. Since, the textile industries have been using a variety of dyes mainly containing 65–75% of azo compounds. It is estimated that during the manufacturing and processing ~12% of these azo dyes are lost per year^3^. The direct discharge of these dyes into the environment is hazardous to both the environment and human beings. MB is not only used as textile azo dye, but it also has large practical applications in various fields, for example in coloring paper, temporary hair dyes, cotton and wool dyes, and medicinal treatments^3^; therefore, it was selected as model dye to evaluate the photocatalytic degradation activity.

## Results and Discussion

**Figure 1:**
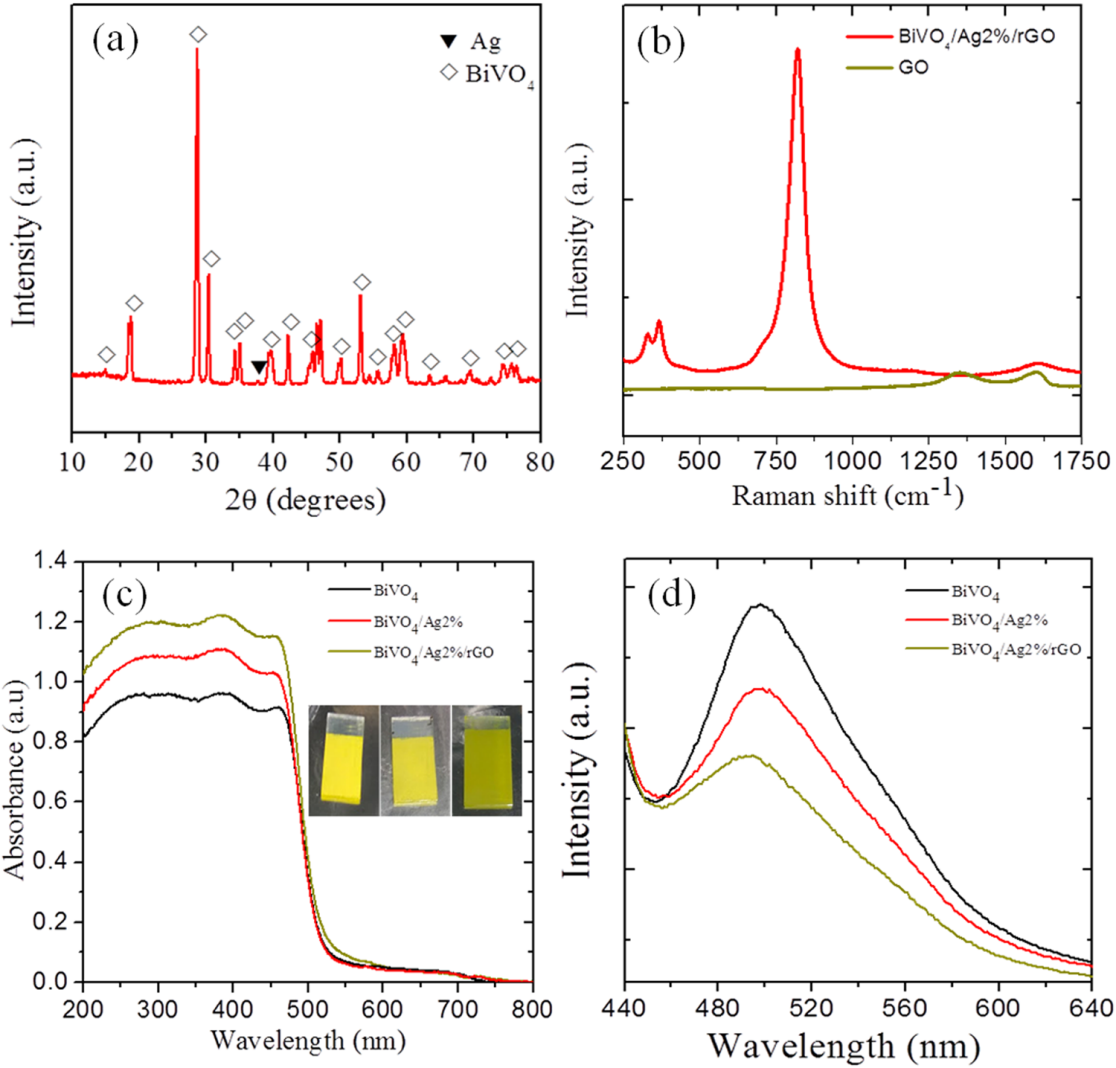
(**a**) XRD pattern of the BiVO_4_/Ag2%/rGO sample. (**b**) Raman spectra of graphene oxide (GO) and the BiVO_4_/Ag2%/rGO sample. (**c**) UV-Vis absorption spectra of various samples. Inset shows the photographs of the BiVO_4_, BiVO_4_/Ag2%, and BiVO_4_/Ag2%/rGO samples. (**d**) PL spectra of various samples.

Figure 1a depicts the XRD pattern of the BiVO_4_/Ag2%/rGO hybrid sample confirming the formation of monoclinic BiVO_4_ (space group 12/a) with lattice constant values of *a* = 5.185 Å, *b* = 11.713 Å, and *c* = 5.102 Å, which is consistent with the standard diffraction pattern of BiVO_4_ (JCPDS card No. 014–0688). An additional weak diffraction peak (~37.5) corresponding to the cubic phase of Ag nanoparticles (JCPDS card no. 04–0783) was observed. No peaks of rGO appeared in the diffraction pattern, which might be due to the weak diffraction intensities or low amount of rGO within the hybrid sample. Additionally, the XRD patterns of bare BiVO_4_ and rGO has been provided for comparison (please see the supplementary information Fig. S1a and b). The Raman structural analysis of the hybrid sample displayed peaks at ~820, 367, 324, and 210 cm^−1^ that could be ascribed to the monoclinic BiVO_4_ (Fig. 1b). The peak at 820 cm^−1^ was attributed to the symmetric and antisymmetric stretching modes of the V-O bonds, while the peaks at ~367 and 324 cm^−1^ were assigned to the distinctive symmetric and antisymmetric bending modes of the vanadate anion. Similarly, the two weak peaks at 1350 and 1600 cm^−1^ represent the distinctive features of the D and G bands of rGO. The Raman spectra of pure BiVO_4_ and Ag was also recorded for comparison (supplementary information Fig. S2). To investigate the optical properties of the samples, UV-Vis absorption spectra were acquired (Fig. 1c). All BiVO_4_ based hybrid samples showed a strong optical absorption in the 500–600 nm wavelength range, therefore they could act as photoanodes/photocatalysts under visible light irradiation. The corresponding band gap energies were estimated from the Tauc plots of all samples and found to be in the range of 2.35–2.42 eV, which are consistent with previous reports^12, 20^ (Supplementary information Fig. S3). Similarly, a weak optical absorption around 400 nm was attributed to the surface plasmon resonance of the Ag nanoparticles. To further investigate the radiative recombination or separation efficiency of the photoinduced charge carriers (e^−^/h^+^), photoluminescence spectra were obtained (Fig. 1d). It is widely understood that a lower PL intensity corresponds to a better charge carrier separation and lower e^−^/h^+^ recombination^27^. As it can be seen in Fig. 1d, a broad characteristic PL band of BiVO_4_ at around 510 nm can be ascribed to the radiative recombination of the charge carriers (e^−^/h^+^). Among all samples, the pristine BiVO_4_ sample showed the highest PL intensity indicating a quicker recombination of e^−^/h^+^, whereas a decreased PL intensity was observed for the Ag loaded hybrid sample (BiVO_4_/Ag2%). This decrease in PL intensity could be due to the interaction between Ag and BiVO_4_ facilitating the e^−^/h^+^ separation through a substantial charge transfer from BiVO_4_ to the Ag NPs, since Ag acts as an electron sink. The PL intensity was further reduced upon incorporation of rGO into the hybrid structure (BiVO_4_/Ag2%/rGO), indicating an enhanced e^−^/h^+^ separation. The superior e^−^/h^+^ separation could be attributed to the heterojunction formation leading to an efficient charge transfer process that will be further discussed in detail.

**Figure 2:**
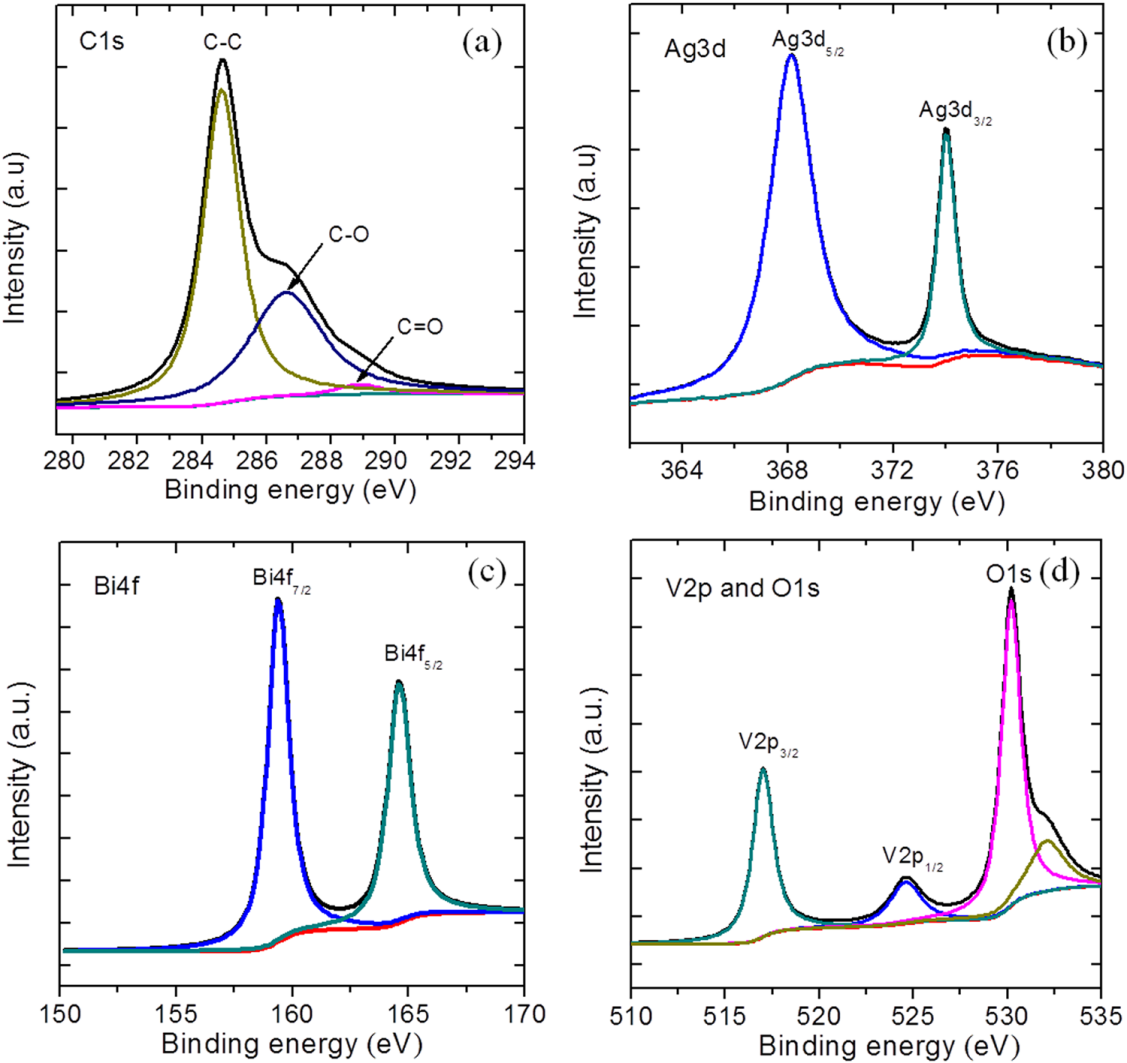
XPS spectra of the BiVO_4_/Ag2%/rGO sample (**a**) C1s, (**b**) Ag3d, (**c**) Bi4f, (**d**) V2p, and O1s, respectively.

The surface composition and elemental states of the hybrid sample were characterized by XPS spectroscopy (Fig. 2), which confirmed that the hybrid sample is composed of the C, Bi, O, V, and Ag elements. The XPS survey spectrum is shown in Fig. S4 (see Supplementary information). The presence of rGO is demonstrated by the appearance of the carbon peaks in the XPS spectrum. The characteristic peaks in the C1s spectrum (Fig. 2a) can be attributed to the graphitic sp^2^ carbon atoms of the C-C species (284.6 eV). Similarly, the other two carbon species, C-OH (286.6 eV) and C=O-H (288.8 eV), could be ascribed to the residual oxygenated groups on the surface of rGO^28^. The XPS spectrum of Ag3d (Fig. 2b) displays two peaks corresponding to two different binding energies, namely Ag3d_5/2_ (368 eV) and 3d_3/2_ (373.9 eV). The binding energy difference of 5.9 eV between the two states of Ag3d confirms the formation of metallic Ag in the as-prepared hybrid sample^4, 29^. Figure 2c displays the XPS spectrum of Bi4f revealing two peaks assigned to Bi 4f_7/2_ (159.0 eV) and Bi 4f_5/2_ (165 eV). Similarly, as shown in Fig. 2d, the split peaks of V 2p correspond to V 2p_3/2_ (524.2 eV) and V 2p_1/2_ (516.8 eV), suggesting the presence of a V^5+^ state in the BiVO_4_ architectures. Figure 2d displays the XPS spectrum of O 1s, indicating an asymmetric behavior of the oxygen states that are deconvoluted into peaks with binding energies centered at 529.9 and 531.6 eV, which can be assigned to the lattice oxygen and surface hydroxyl groups of BiVO_4_, respectively.

**Figure 3:**
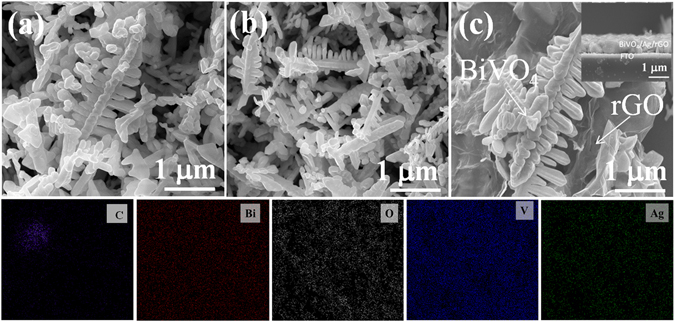
FESEM micrographs of (**a**) BiVO_4_, (**b**) BiVO_4_/Ag2%, and (**c**) BiVO_4_/Ag2%/rGO hybrid architectures and the corresponding EDS mapping images.

The surface morphological features of the samples were investigated by using field emission scanning microscopy (FE-SEM). Figure 3a–c shows the FE-SEM micrographs of pristine BiVO_4_, BiVO_4_/Ag, and BiVO_4_/Ag/rGO hybrid samples, respectively. BiVO_4_ exhibited a unique fern/dendritic hyperbranched surface morphology (Fig. 3a). It is worth to mention that special fern/dendritic architectures have gained the attention of researchers due to the remarkable connectivity between their crystals, which led to improved electrochemical and catalytic properties^28, 30, 31^. Submicron sized BiVO_4_ fern architectures were formed by perpendicular alignment of the several interconnecting rod-like sub-branches (300–500 nm) supported by an intact backbone (5–7 μm). Upon hydrothermal reaction, the Brownian motion or short-range interaction of particles favors the growth of rod-like structures. The as-obtained rod-like structures further grow according to well-known self-assembly and Ostwald ripening growth mechanisms to adjust the minimum total surface free energy, thereby developing into particular dendritic/fern architectures. A previous study also claims that an isotropic growth of rod-like structures in the [001] direction accounts for the formation of unique BiVO_4_ dendritic/fern architectures^28, 32^. The possible chemical reactions and growth mechanisms during the hydrothermal reaction are illustrated in Fig. S5 (see Supplementary information S2). The EDS mapping image of the BiVO_4_/Ag2%/rGO hybrid sample was obtained (Inset) and displays C, Bi, O, V, and Ag as the constituent elements of the hybrid sample.

**Figure 4:**
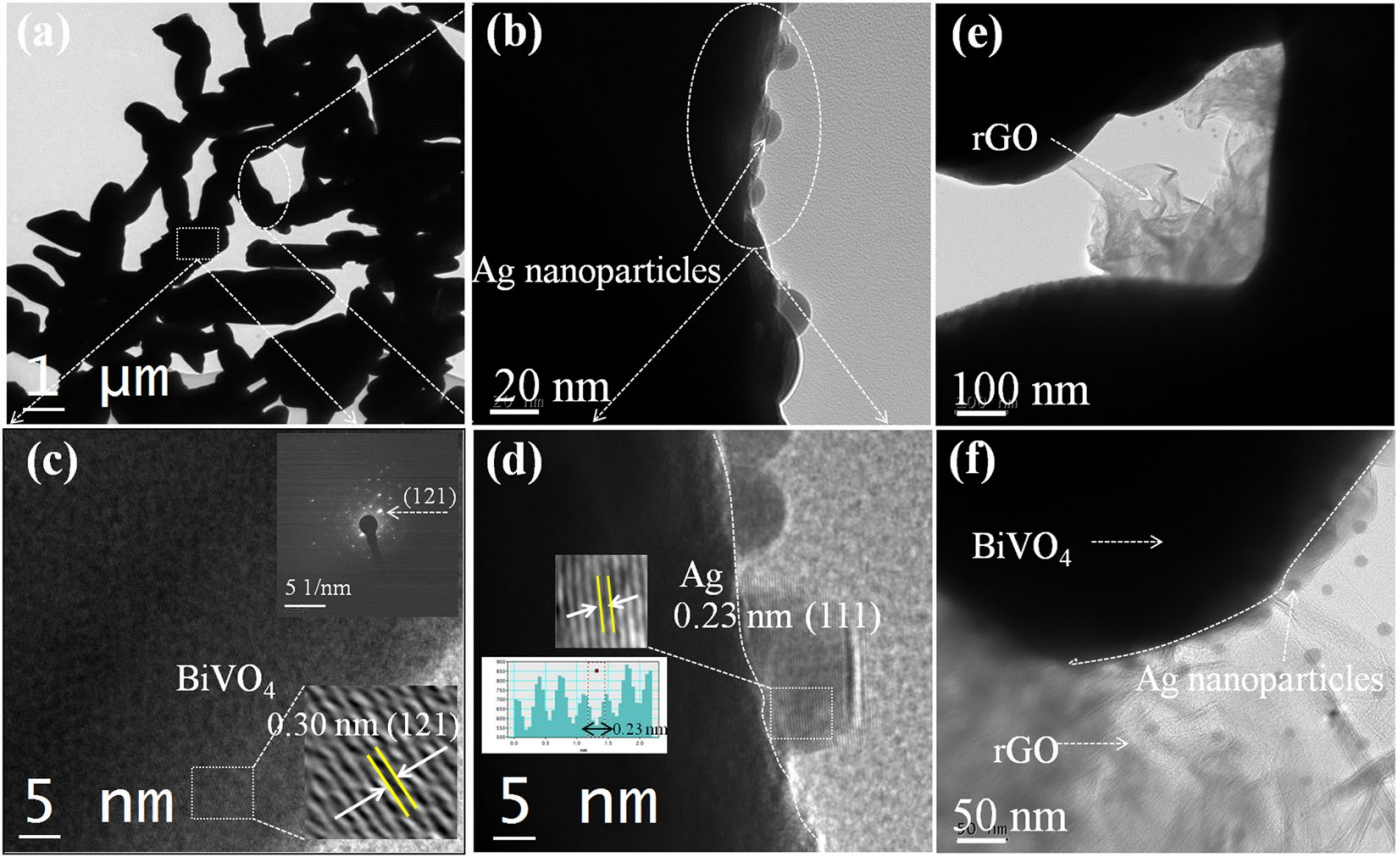
TEM and HRTEM images of (**a**–**d**) BiVO_4_/Ag2% hybrid sample (inset shows the SAED pattern) and (**e**,**f**) BiVO_4_/Ag2%/rGO hybrid sample.

Next, the particle size and crystal structure were determined by using high resolution transmission electron microscopy (HRTEM) that provided results consistent with those of the earlier FESEM micrographs. Figure 4a–d represents the TEM and HRTEM micrographs of the hybrid sample (BiVO_4_/Ag2%), indicating submicron sized BiVO_4_ dendritic structures that are composed of a 5–7 μm long intact backbone structure and 300–700 nm sized rod-like sub-branches. Ag NPs with a size around 15–20 nm were observed in the vicinity of BiVO_4_, forming an interface with BiVO_4_. The TEM image was partly magnified into separate figures (Fig. 4b,c and d) to illustrate the intimate contact and interface between the hybrid structures. The interface is shown by a dotted white colored curved line. As it can be seen in Fig. 4b, Ag nanoparticles with sizes of ~15–20 nm are present on the surface of BiVO_4_. The HRTEM micrographs (Fig. 3c and d) clearly showed two distinct sets of lattice fringes with interplanar lattice spacings of 0.30 and 0.237 nm, which correspond to the (121) and (111) crystallographic planes of BiVO_4_ and Ag NPs, respectively. The occasional bright spots in the selected area electron diffraction (SAED) pattern (inset of Fig. 4c) coincide with the (121) crystallographic plane of monoclinic BiVO_4_. As shown in Fig. 4e and f, it is evident that both rGO thin sheets and Ag NPs do exist in the hybrid sample, supporting the BiVO_4_/Ag2%/rGO heterostructure formation. The EDS analysis (Supplementary information Fig. S6) confirmed the presence of the C, Bi, Ag, V, and O elements in the as-prepared hybrid sample.

**Figure 5:**
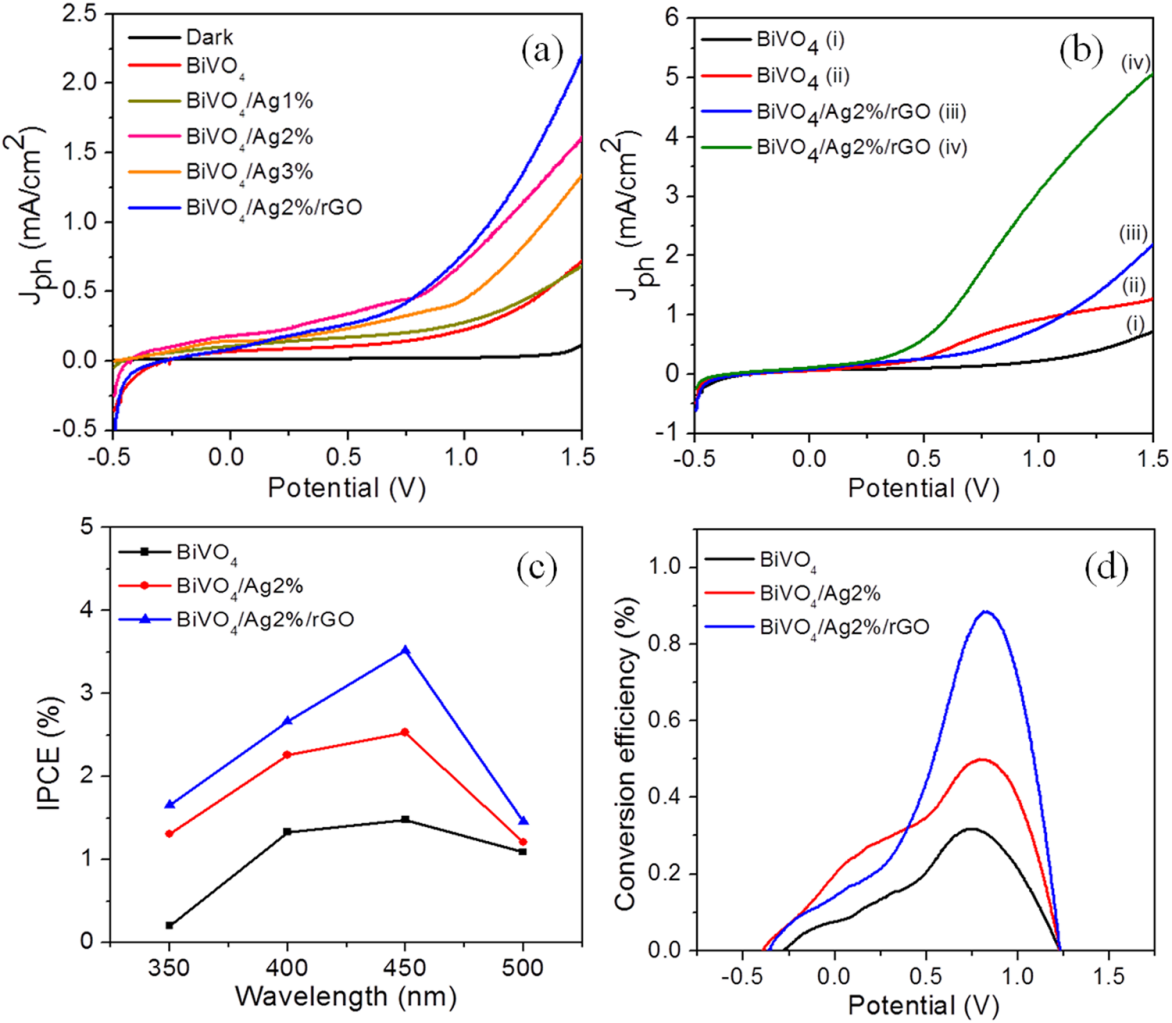
(**a**) Photocurrent density-voltage (J-V) characteristics of the samples under dark and illumination conditions using 0.5 M Na_2_SO_4_ as electrolyte solution. (**b**) Comparative J-V curves of the BiVO_4_ sample (i, ii) using 0.5 M Na_2_SO_4_ and 0.5 M Na_2_SO_3_ as electrolyte, respectively, and (iii, iv) J-V curves for the BiVO_4_/Ag/rGO sample using 0.5 M Na_2_SO_4_ and 0.5 M Na_2_SO_3_ as electrolyte, respectively. (**c**) Solar to hydrogen conversion (STH) efficiency. (**d**) Incident photon to current conversion efficiency (IPCE) calculated from wavelength dependent current voltage measurements.

To evaluate the photoanodic behavior of BiVO_4_ based hybrid electrodes, a systematic PEC study was carried out with simulated sunlight illumination (AM 1.5 G) in an aqueous electrolyte (0.5 M Na_2_SO_4_ and 0.5 M Na_2_SO_3_) using a regular two-electrode homemade PEC cell (Supplementary information Fig. S7). An important performance metric of the water splitting photoelectrodes is the stability of the photocurrent under light illumination, which directly correlates to the oxygen evolution rate^4, 33^. The effect of Ag and Ag/rGO on the PEC properties of the BiVO_4_ nanostructures was examined. Figure 5a shows the linear sweeps recorded for the pristine BiVO_4_ and BiVO_4_ hybrid photoanodes in 0.5 M Na_2_SO_4_ electrolyte at a scan rate of 10 mV/s. It can be seen that the linear sweep curves under dark conditions were negligible for all the photoanodes even at high applied potential, embodying the improved quality and stability of the photoanodes. On the other hand, under light illumination the photocurrent of pure BiVO_4_ (red curve) increased linearly with increasing applied potentials. The BiVO_4_ photoanode exhibited a noticeable photocurrent density (*J*_ph_) of 0.5 mA cm^−2^ at an applied bias (1.5 V *vs* RHE). Similarly, the BiVO_4_ hybrid photoanodes showed a qualitatively similar behavior to that of BiVO_4_, while exhibiting a much more significant enhancement of the *J*_ph_ values. The *J*_*ph*_ values were found to be improved upon substantial Ag loading on the BiVO_4_ structures. The maximum *J*_*ph*_ of 1.7 mA/cm^2^ was observed for an Ag2% loading that is about 3 times higher than that of the pristine BiVO_4_ photoelectrode. A further increase of the Ag loading (Ag > 2 wt.%) onto the BiVO_4_ photoelectrode led to a lowered *J*_*ph*_ value (~1.3 mA/cm^2^), as evidenced by the BiVO_4_/Ag3% sample. The enhancement of the BiVO_4_ photocurrent at an optimal Ag loading could be attributed to three main factors: (i) improved electrical conductivity of BiVO_4_, (ii) increased charge separation efficiency of the photoinduced e^−^/h^+^ pairs, and (iii) reduced charge carrier resistance or charge recombination rate ascribed to the BiVO_4_ and Ag Schottky barrier formation and interaction^34^. Additionally, it has been reported that the LSPR effect of Ag aids to the solar water splitting utilizing the plasmon resonance energy transfer^26^. Although high photocurrent densities were obtained for the Ag loaded BiVO_4_ samples, the values were disappointingly lower than the expected theoretical values. To further improve the PEC-WS performance of the optimal BiVO_4_/Ag2% sample, a structural modification approach based on carbonization was adopted using reduced graphene oxide. As illustrated in Fig. 5a (blue curve), the rGO modified BiVO_4_/Ag2% photoelectrode (BiVO_4_/Ag2%/rGO) exhibited a maximum *J*_ph_ of ~2.25 mA/cm^2^, which is about 4.5 and 1.32 times higher than that of the pristine BiVO_4_ (0.5 mA/cm^2^) and binary BiVO_4_/Ag2% (1.7 mA/cm^2^) hybrid photoanodes, respectively.

The PEC characteristics of the as-prepared photoanodes were further examined in the presence of a 0.5 M sodium sulfite (Na_2_SO_3_) electrolyte, which served as hole scavenger. The sulfite oxidation is thermodynamically and kinetically more favored over the oxidation of water^19^; hence, the photocurrent measurement for the sulfite oxidation enables the investigation of the photoelectrochemical properties of BiVO_4_, despite its poor water oxidation kinetics^6, 35^. Figure 5b shows a comparative study of the *J*-*V* curves of the BiVO_4_ and hybrid BiVO_4_/Ag2%/rGO photoanodes in 0.5 M Na_2_SO_4_ and 0.5 M Na_2_SO_3_ electrolytes, respectively. The *J*-*V* curves for the hybrid BiVO_4_/Ag2%/rGO sample in both electrolytes showed a qualitatively similar behavior as a function of the potential. However, the overall *J*_*ph*_ was significantly higher for the sulfite oxidation (using 0.5 M Na_2_SO_3_) than for the water oxidation (using 0.5 M Na_2_SO_4_), suggesting that the rates of charge carrier generation, separation, and transportation were improved confirming that the oxidation at the photoanode surface was not rate limiting, since Na_2_SO_3_ acted as a hole scavenger. An overall enhancement of the photocurrent was also observed for the pristine BiVO_4_ and other BiVO_4_ hybrid photoanodes in the sulfite oxidation (see Supplementary information Fig. S8). At ~1.5 V applied bias, the *J*_*ph*_ obtained for BiVO_4_/Ag2%/rGO for the sulfite oxidation was ~5 mA/cm^2^, which is about 1.5 times higher than that of BiVO_4_/Ag2%/rGO photoelectrode for the water oxidation. The obtained values are comparable with the recently reported BiVO_4_ based photoanodes (see Supplementary information Table S1). Thus, the improvement of the *J*_*ph*_ of the BiVO_4_/Ag2%/rGO hybrid photoelectrode unambiguously suggests that the hybridization of the BiVO_4_ structures with the Ag NPs and conducting two-dimensional rGO thin sheet structures results to be quite beneficial for PEC applications. Our analysis of the BiVO_4_/Ag/rGO hybrid photoanode reveals that the Ag NPs effectively improve the charge carrier separation and bulk electronic conductivity of BiVO_4_, while rGO further assists the transportation of the surface charge carriers resulting in an enhanced light absorption and PEC-WS efficiency under simulated sunlight illumination. It is also widely accepted that the higher surface area is useful to generate more active sites and light harvesting which is useful for better PEC performance^27^. The BET surface area was measured from the N_2_ adsorption-desorption isotherms (Supplementary information Fig. S9). The BiVO_4_/Ag2%/rGO photocatalyst sample showed the surface area of 16.35 m^2^/g which is relatively higher than that of BiVO_4_ (9.44 m^2^/g) and BiVO_4_/Ag2% (8.76 m^2^/g).

To investigate the enhanced light absorption, the incident photon-to-current conversion efficiency (IPCE) under monochromatic light irradiation was determined for the as-prepared photoanode samples. The IPCE was calculated at an applied potential of 0.1 V according to Eq. 1:

1 $$IPCE( \% )=\frac{1240(V\times nm)\times {J}_{ph}(mAc{m}^{-2})}{\lambda (nm)\times {P}_{light}(mWc{m}^{-2})}\times 100$$

where the parameter 1240 stands for the multiplication of the speed of light and Planck’s constant, *λ* is the wavelength of the monochromatic photons, *J*_*ph*_ is the produced photocurrent density, and *P*_*light*_ is the power density of the light at a given wavelength. As shown in Fig. 5c, at the monochromatic wavelength of 450 nm, the highest value of IPCE of ~3.5% was obtained for the BiVO_4_/Ag2%/rGO hybrid photoelectrode, which is approximately 2.3 and 1.4 folds higher than that of the pristine BiVO_4_ and BiVO_4_/Ag2% photoelectrodes, respectively. The poor IPCE confirms the low photoactivity of the bare BiVO_4_ due to its scarce charge carrier mobility. The IPCE slightly improved after Ag loading, while increasing significantly when both rGO and Ag were integrated into the BiVO_4_ structures. This indicates that both Ag and rGO nanostructures contribute to the enhancement of the light absorption properties; therefore, the fabrication of the hybrid BiVO_4_/Ag2%/rGO electrode enables high conversion efficiencies as well as better charge carrier mobility^36^.

Subsequently, in order to check the ability of the as-prepared photoanode for artificial photosynthesis, the solar to hydrogen conversion efficiency (STH %) was evaluated (Fig. 5d) by using Eq. 2:

2 $$STH( \% )=\frac{{J}_{ph}(mAc{m}^{-2})\times (1.23-|{V}_{app}|)(V)}{{P}_{light}(mWc{m}^{-2})}\times 100$$

where *J*_ph_ is the measured photocurrent density, 1.23 V is the standard state reversible potential of water, |*V*_*app*_| = (*V*_*meas*_ − *V*_*oc*_), *V*_*meas*_ is the applied potential during the measurement of the photocurrent density, *V*_*oc*_ is the open circuit potential of the working electrode, and *P*_*light*_ *t* is the illuminating light power density. The measured STH efficiency of the BiVO_4_/Ag2%/rGO hybrid sample was estimated to be 0.9%, which is markedly higher than that acquired for the pristine BiVO_4_ (0.3%) and BiVO_4_/Ag2% (0.5%) photoelectrode samples. The measured STH efficiencies of the hybrid samples seem to be consistent with that of the natural photosynthesis^36^. The improved STH efficiency of the BiVO_4_/Ag2%/rGO hybrid sample again reflects a more efficient sunlight utilization, charge separation and transportation. The Mott-Schottky (M-S) plots were acquired for BiVO_4_ and BiVO_4_/Ag/rGO photoanode samples indicating the flat band potentials of approximately 0.10 V versus RHE and 0.13 V versus RHE, respectively which is consistent with the earlier reports (supplementary information Fig. S10)^6, 37^. Similarly, to elucidate the charge transfer kinetics of the bare BiVO_4_ and hybrid samples, electrochemical impedance spectra were recorded (Supplementary information Fig. S11). Generally, the arcs in the Nyquist plots are considered to confirm the charge transfer characteristics at the photoelectrode/electrolyte interface^12^. As illustrated in Fig. S11, the smaller arc radius of the BiVO_4_/Ag2%/rGO hybrid photoelectrode compared to that of the pristine BiVO_4_ photoelectrode clearly suggests a lower electron transfer resistance at the photoelectrode/electrolyte interface^12, 38^. To analyze the impedance data the equivalent circuit was constructed where *R*_S_ indicates the solution resistance, *R*_ct_ is the charge transport resistance, *W* is Warburg impedance and *Q* represents the constant phase element (CPE). The as obtained fitted values of *R*_s_ for BiVO_4_, BiVO_4_/Ag2% and BiVO_4_/Ag2%/rGO photoelectrodes were found to be 17.28, 12.92 and 9.83 Ω cm^−2^, respectively. Similarly, the fitted values for *R*_ct_ were 128160, 16536 and 9818 Ω cm^−2^, respectively. The lowered values of resistances for BiVO_4_/Ag2%/rGO photoelectrode clearly indicates improved charge carrier transportation and the kinetics in the PEC water-splitting device. The impedance result of the BiVO_4_/Ag2%/rGO hybrid photoelectrode agrees well with the previous PL spectra, which demonstrated that a lower PL intensity is an indication of the longer life time of the photoexcited charge carriers due to a better charge separation and thereby, a lower charge transfer resistance. Moreover, the stability of the photoanode is indispensable to achieve a high efficiency of the PEC-WS devices. The stability of the pristine BiVO_4_ and BiVO_4_/Ag2%/rGO hybrid photoanode samples was tested performing chronoamperometric *J*-*T* curve measurements by applying a small bias voltage of approximately 0.5 V to prevail the further ohmic losses in the electrolyte and metal contacts under simulated light illumination for 10000 sec (Supplementary information Fig. S12). Interestingly, a reasonable photocurrent stability retention over a prolonged time was noticed for the hybrid BiVO_4_/Ag2%/rGO photoanode suggesting that a strategy based on hybrid photoanode fabrication could also help to improve the samples PEC performance against corrosion.

**Figure 6:**
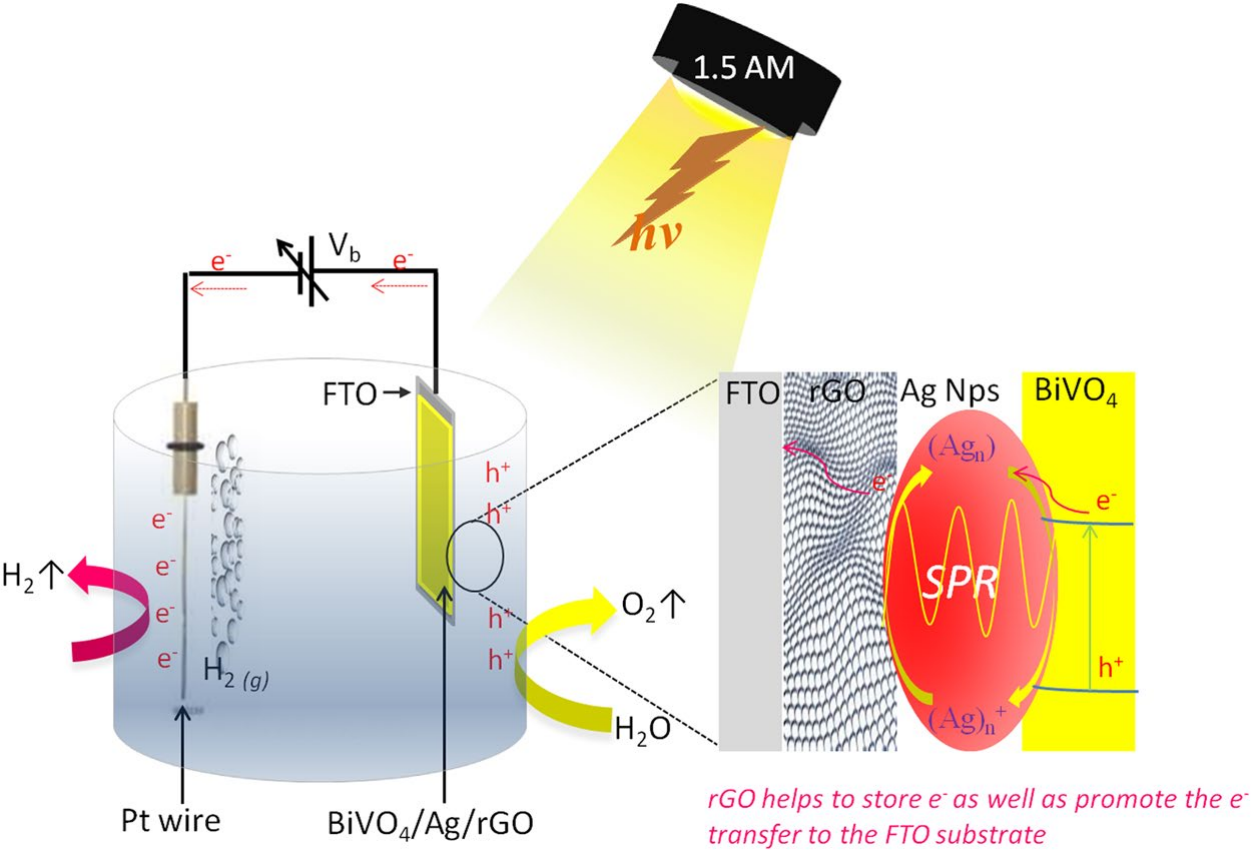
Schematic representation of photoelectrochemical water splitting using the BiVO_4_/Ag/rGO hybrid photoanode sample.

To better understand the photocatalytic reaction mechanism, a schematic representation of PEC-WS using the ternary BiVO_4_/Ag/rGO hybrid photoanode is illustrated in Fig. 6. It can be seen that the illumination of the photoanode is always accompanied with high density e^−^/h^+^ pairs, which usually separates at the semiconductor/electrolyte interface due to the band bending to perform half-cell reactions^39^. In the present case, when the BiVO_4_/Ag2%/rGO photoanode is exposed to light irradiation, both BiVO_4_ and Ag simultaneously absorb the visible light, thereby generating charge carriers (e^−^/h^+^). Moreover, due to the difference in work function between BiVO_4_ and Ag NPs, a Schottky barrier is formed at the interface that could facilitate the separation of the e^−^/h^+^ pairs^12^. The presence of Ag allows a fast transfer of the photoinduced electrons from the conduction band of BiVO_4_ to the electropositive Ag ions^12, 40^, which are then shuttled to rGO since the plasmonic metals have an exceptional ability to accept and shuttle electrons^41, 42^. As a result, a facile charge separation takes place, while the charge recombination is suppressed. Furthermore, rGO contributes to the e^−^ transportation to the conducting FTO coated glass substrate, since rGO possesses a unique electronic structure that can store and transport electrons to the conducting substrate very easily as required^38^. Eventually, the electrons start to migrate from the FTO conducting substrate to the Pt counter electrode, wherein they react with hydrogen ions producing molecular H_2_. Meanwhile, the holes are drifted to the photoanode surface, wherein they are utilized to remove the oxygen from the water. Therefore, an enhanced light absorption and effective e^−^/h^+^ separation, transportation, and capture by the targeted molecules may take place due the synergistic effect of the Schottky barrier formation, localized surface plasmon resonance, and improved electrical properties in the BiVO_4_/Ag/rGO hybrid structure, thus enhancing the PEC-WS performance.

**Figure 7:**
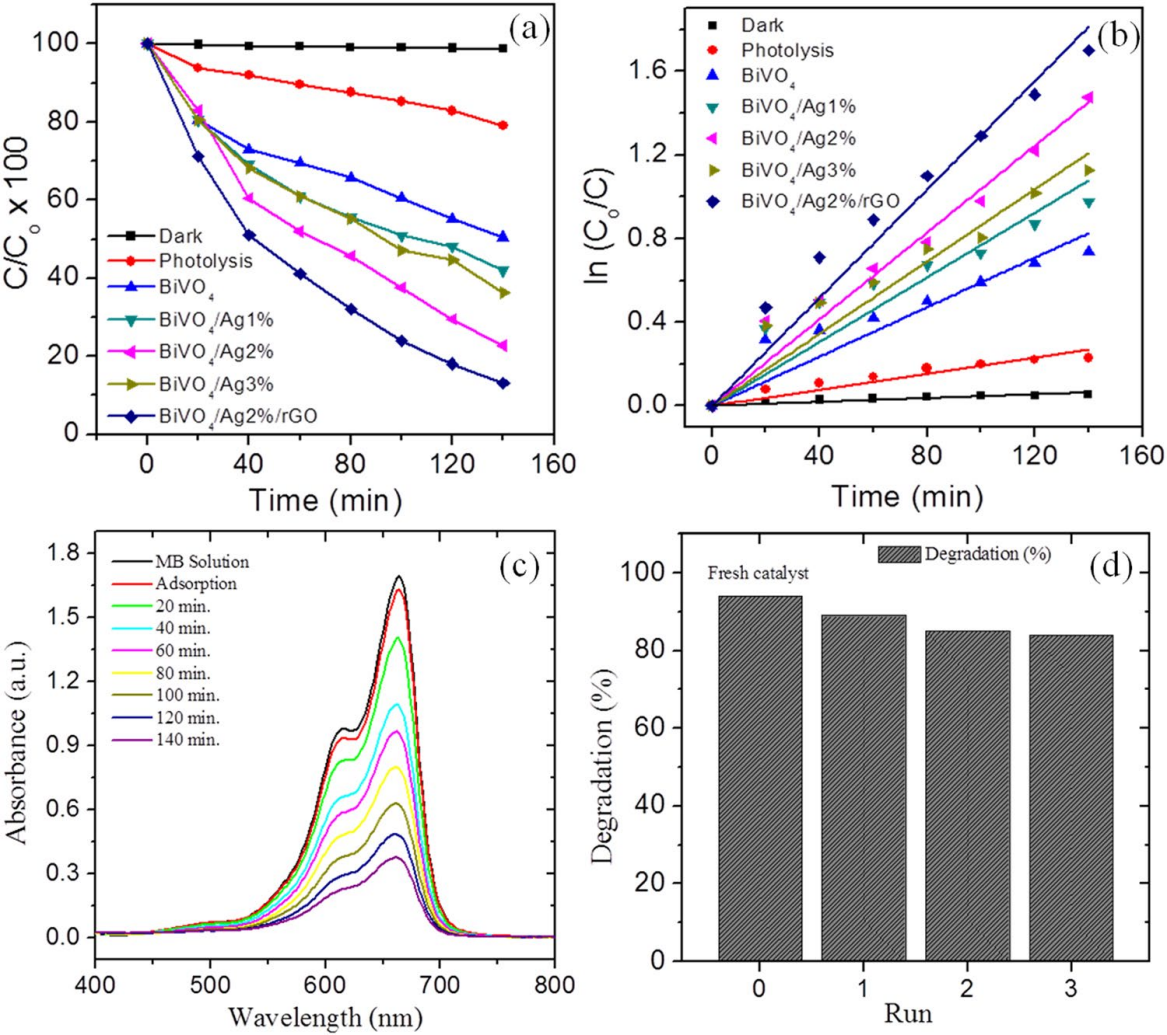
(**a**) Degradation of MB with time using different samples under visible light irradiation. (**b**) Chemical kinetic study of photocatalytic MB degradation over various samples. (**c**) UV-Vis absorption spectra of the MB dye solution during photocatalysis at successive time intervals in the presence of the recycled BiVO_4_/Ag2%/rGO photocatalyst sample. (**d**) Bar diagram of the recycled runs showing the MB dye degradation efficiency over the BiVO_4_/Ag2%/rGO sample.

Since the hybrid BiVO_4_ heterostructures showed a good PEC performance owing to their superior charge separation and transfer, we also tested the as-prepared BiVO_4_ hybrid photocatalyst samples (in their powder form) against the photocatalytic degradation of the organic dye pollutant methylene blue (MB) under visible light irradiation (λ > 420 nm) without the use of any sacrificial agent. Figure 7a depicts the photocatalytic MB degradation efficiency over different photocatalyst samples. The apparent rate constant (*k*) values for the MB degradation were calculated from the slope of the plot of ln(*C*_*o*_ */C*) versus irradiation time, where *C*_*o*_ is the initial MB concentration and *C*_*t*_ is the concentration of MB at the time *t*, and are summarized in Table S2 (see Supplementary information). It can be understood that the negligible photocatalytic activity was observed for the MB degradation under dark conditions (Fig. 7a and b). On the other hand, under light irradiation conditions, the photocatalytic MB degradation performance for as synthesized photocatalysts was drastically enhanced. A rate constant of k = 1.29 × 10^−2^ min^−1^ was observed for the BiVO_4_/Ag2%/rGO hybrid photocatalyst, which is notably higher than that of the pristine BiVO_4_ (0.59 × 10^−2^ min^−1^), BiVO_4_/Ag1% (0.76 × 10^−2^ min^−1^), BiVO_4_/Ag2% (1.03 × 10^−2^ min^−1^), and BiVO_4_/Ag3% (0.86 × 10^−2^ min^−1^) samples. The highest MB degradation rate constant of *k* = 1.29 × 10^−2^ min^−1^ was observed for the hybrid BiVO_4_/Ag2%/rGO photocatalyst sample, and it is about ~2.18 and ~1.25 folds higher than that of the pristine BiVO_4_ and BiVO_4_/Ag2% photocatalysts, in agreement with the PEC results. Additionally, in order to determine the fundamental role of the main reactive species involved in the photocatalytic MB degradation the scavengers such as isopropyl alcohol (IPA) and benzoquinone (BQ)^43, 44^ were added (10 mL) to the photocatalytic reaction prior to the irradiation (please see the supplementary information Fig. S13). It was revealed that the addition of scavengers resulted into the decreased degradation rate constant from *k* = 1.29 × 10^−2^ min^−1^ (no scavenger) to *k* = 0.43 × 10^−2^ min^−1^ (IPA) and k = 0.30 × 10^−2^ min^−1^ (BQ), suggesting that the holes (h^+^) are the effective reactive species for MB degradation^45^.

Furthermore, a colorless organic pollutants (phenol) was also chosen to evaluate the photocatalytic activity of BiVO_4_/Ag2%/rGO photocatalyst sample. The degradation of phenol was performed under the similar reaction condition as that of MB and the absorption spectrum was monitored at regular intervals of time (Supplementary information Fig. S14). From the UV-visible absorption spectra, the pseudo first order degradation rate constant was calculated for the phenol degradation which is found to be 0.196 × 10^−2^ min^−1^ The XPS spectrum for the recycled sample was acquired to examine the presence and the state of Ag after photocatalysis. The XPS spectrum of Ag showed no obvious change or shift in the spectrum confirming the stability of photocatalyst (supplementary information Fig. S15). It is also important to note that an insignificant deactivation of the photocatalyst (BiVO_4_/Ag2%/rGO) or loss of photocatalytic activity was observed, even after three consecutive photocatalytic cycles, indicating an improved stability of the prepared hybrid samples (Fig. 7c). The photocatalytic activity of the recycled BiVO_4_/Ag2%/rGO hybrid photocatalyst sample is shown by a bar diagram (Fig. 7d) indicating a consistent MB degradation efficiency.

## Conclusion

This study highlighted the feasibility of a facile and cost effective synthesis of photoanodes by a hydrothermal method to incorporate/integrate Ag and rGO into BiVO_4_ nanostructures, and investigated their PEC-WS and photocatalytic MB degradation performances. The resulting BiVO_4_/Ag/rGO hybrid photoanode demonstrated a significant improvement of the PEC-WS performance, which was about 4.5 times higher than that of pristine BiVO_4_; it also exhibited a higher IPCE of ~3.5% and STH of ~0.9%, indicating a significant light harvesting and charge transport characteristics. Similarly, a high apparent rate constants (kapp) of 1.29 × 10^−2^ and 0.192 × 10^−2^ was achieved for the photocatalytic MB and phenol degradation, respectively using the BiVO_4_/Ag/rGO hybrid architectures, consistent with the aforementioned PEC-WS results. The notable enhancement of the PEC/photocatalytic properties can be attributed to the synergistic effect of the incorporated Ag and rGO nanostructures, which contributes to the interface formation wherein the efficient separation, transportation, and utilization of the charge carriers (e^−^/h^+^) may occur. From a practical point of view, the present synthetic strategy could be employed for the development of cost effective semiconductor heterostructure photoanodes. Furthermore, the present BiVO_4_/Ag/rGO hybrid architectures are promising candidates for use in photovoltaics.

## Methods

### Synthesis of graphene oxide (GO)

Graphene oxide was synthesized according to a modified Hummers method as previously reported using commercial graphite powder (Sigma-Aldrich)^46^. For the exfoliation, 1 g of graphite was added to concentrated H_2_SO_4_ (23 mL) in a round-bottom flask. The mixture was magnetically stirred upon cooling in an ice bath for 5 min. Then, 3 g of KMnO_4_ powder was slowly added in small quantities under vigorous stirring and maintained at 20 °C for 10 min. Subsequently, the solution was allowed to stir for 4 h and the temperature was maintained at 35 °C, until the formation of a dark brown colored paste. Distilled water (46 mL) was added to the aforementioned brown paste, the reaction temperature was gradually raised to 98 °C, and the stirring was continued for another 15 min. To the reaction mixture, 140 mL of distilled water and 10 mL of H_2_O_2_ (30%; Fisher Scientific) solution were then added. Finally, the precipitate was washed thoroughly with 5% HCl (38%, SDFCL) and collected by centrifugation. The as-obtained GO powder was dried overnight in an oven, characterized, and used for further reactions (see Supplementary information Fig. S16, for characterization).

### Synthesis of BiVO_4_/Ag/rGO hybrid heterostructures

The BiVO_4_/Ag/rGO photoanode samples were synthesized by a simple and surfactant free *in situ* hydrothermal method. Thus, 5 mL of nitric acid (HNO_3_, 70%; OCI company Ltd.), 55 mL of distilled H_2_O, and 5 mL of ethanol (C_2_H_5_OH, 94.5%; DAEJUNG) were placed in a beaker and mixed for 5 min under magnetic stirring. Then, 1.12 mmol of bismuth (III) nitrate (Bi(NO_3_)_3_.5H_2_O, 98%; DAEJUNG) and 1.12 mmol of ammonium metavanadate (NH_4_VO_3_, 99%; DAEJUNG) were added to the above solution and kept under magnetic stirring until all precursors were dissolved. An appropriate amount of silver nitrate AgNO_3_, (99.5%; EMPARTA ACS) was added to the above reaction solution and magnetically stirred for 5 min. Then, an ammonia solution (NH_4_OH, 25%; DAEJUNG.) was slowly added to the reaction mixture, which was allowed to stir for 10 min, while the pH was maintained at ~3–3.5. Meanwhile, 30 mL of a GO dispersion (0.5 mg/mL) in distilled water was ultrasonicated for 3 h to exfoliate the graphene sheets, and then added to the aforementioned reaction mixture. The reaction solution was ultrasonicated for another 10 min. Subsequently, the FTO coated glass substrates were cleaned under ultrasonication using acetone, ethanol, and distilled water. The clean FTO substrates were placed in a hydrothermal reactor with the conducting surface of the glass facing down and with an angle of approximately ~45° to the wall of the Teflon vessel. Then, the reaction solution was transferred into a Teflon rector fitted into a stainless steel autoclave and kept in an oven at 150 °C for 12 h. The hydrothermal reactor was allowed to cool naturally to room temperature. This process enabled us to fabricate the BiVO_4_/Ag/rGO thin film photoanodes for use in the PEC-WS cell. A powder collected in the hydrothermal reactor bed was washed thoroughly with distilled H_2_O and C_2_H_5_OH, dried in an oven at 60 °C for 3 h, and used for further characterization and application.

### Photoelectrochemical (PEC) water splitting

The photoelectrochemical water splitting experiments were performed using a homemade PEC cell constituted of a Teflon chamber. PEC-WS measurements were carried out using 0.5 M Na_2_SO_4_ (pH-7) and 0.5 M Na_2_SO_3_ (pH-6.8) as aqueous electrolytes in a two-electrode configuration connected to a PARSTAT 3000 high polarization potentiostat. The as-synthesized BiVO_4_ based hybrid electrodes were used as the photoanode, whereas a Pt wire was used as counter electrode. The photoanode was mounted on the Teflon chamber with an O-ring (surface area of approximately 0.52 cm^2^) in direct contact with the electrolyte solution. A quartz window was fixed on the front side of the Teflon chamber, through which a 300 W xenon lamp light was passed, and the photoanode was exposed to a light irradiation of approximately 100 mW/cm^2^. Further, a set of band-pass filters with specific wavelength values were used to alter the wavelength of the illuminating light in order to carry out the IPCE measurements. The photograph of photoelectrode samples after PEC-WS analysis was taken which indicates the good adhesion and the quality of the film (Supplementary information Fig. S17)

### Photocatalytic methylene blue (MB) degradation

The methylene blue dye was chosen as a model pollutant to evaluate the photocatalytic activity. The photocatalytic degradation of the MB dye at a concentration of 10 ppm was performed in an aqueous solution using a 300-W Xe lamp (λ ≥ 420 nm). The photocatalytic process was performed in a custom water-cooled borosilicate glass reactor capable of maintaining the photochemical reaction temperature at ~25 °C. A photocatalyst sample of ~10 mg was dispersed in an aqueous solution of MB (50 mL) for each irradiation test. Prior to irradiation, the suspension was magnetically stirred under dark conditions for 90 min to establish an adsorption-desorption equilibrium between the photocatalyst and MB solution. The photochemical reaction was carried using a visible light source under magnetic stirring. Aliquots of 4 mL were drawn and filtered at regular time intervals (20 min). The main absorption (~664 nm) of the MB concentration in the filtrate was measured using UV–Vis absorption spectroscopy.

### Characterization

The phase determination of the samples was carried out by using a XRD PANalytical X’Pert PRO system (Netherlands). The Raman spectra were recorded using a Raman microscope (LabRam HR8000 UV, (Horiba Jobin-Yvon, France), KBSI, Gwangju center) with an excitation wavelength 514 nm. The morphological features of the as-prepared samples were investigated using a field emission scanning electron microscope (FE-SEM, Model Hitachi S 4800, Japan, KBSI, Gwangju center) attached to an energy dispersive X-ray spectroscopy (EDS) analyzer to measure the sample composition. The microstructure investigation was carried out by high-resolution transmission electron microscopy (HR-TEM) using a JEOL-3010 at an operating voltage of 300 kV. The chemical binding energy of the samples was measured using a high-resolution X-ray photoelectron spectroscope (HR-XPS, VG Multi lab 2000, Thermo VG Scientific, UK) at room temperature. Optical absorption spectra were obtained using a UV–Vis spectrometer (Lambda 950 spectrometer, Perkin-Elmer). The band gap values were estimated from the graph of (*αhv*)^1/2^ versus photon energy (*hv*) by Kubelka-Munk function indicating the relation between band gap and absorption coefficient. The equation can be expressed as *αhv* = A (*hv* − *E*_*g*_)^1/2^, where *α*, *v* and *h* represents the absorption coefficient, frequency of the light and Planck’s constant, respectively. Photoluminescence spectra were obtained by using a Shimadzu (RF-5301 PC) spectrophotometer with an excitation wavelength of 350 nm. The Brunauer-Emmett-Teller (BET) specific surface areas of the samples were measured based on nitrogen adsorption and desorption isotherms using BELSORP-mini II (BEL, Japan) instrument.

## Electronic supplementary material

Supplementary Information
