# Supplementary material for: One-Pot in Situ Hydrothermal Growth of BiVO4/Ag/rGO Hybrid Architectures for Solar Water Splitting and Environmental Remediation
Source: Sci Rep. 2017 Aug 21;7:8404. doi: 10.1038/s41598-017-08912-z (PMC5566408; doi:10.1038/s41598-017-08912-z)
Supplement: Supplementary file 1 — Supplementary Information [file 41598_2017_8912_MOESM1_ESM.pdf]

Supplementary Information

One Pot in Situ Hydrothermal Growth of $BiVO_4$/Ag/rGO Hybrid Architectures for Solar Water Splitting and Environmental Remediation

Santosh S. Patil,[a] Mukund G. Mali,[b] Mostafa Afifi,[a] Deepak R. Patil,[c] Sanjay S. Kolekar,[d] Sang-Wan Ryu*[a]

[a] Department of Physics, Chonnam National University, Gwangju 500-757, Republic of Korea

[b] School of Chemical Sciences, Solapur University, Solapur, MS, India

[c]Centre for Materials for Electronics Technology (C-MET), Department of Electronics and Information Technology (DeitY), Govt. of India, Pune, India

[d]Analytical Chemistry and Material Science Laboratory, Department of Chemistry, Shivaji University, Kolhapur, India

CORRESPONDING AUTHOR FOOTNOTE

*Prof. Sang-Wan Ryu

Department of Physics, Chonnam National University, Gwangju 500-757, Republic of Korea

Email address: sangwan@chonnam.ac.kr

S1: XRD patterns of $BiVO_4$ and reduced graphene oxide (rGO).

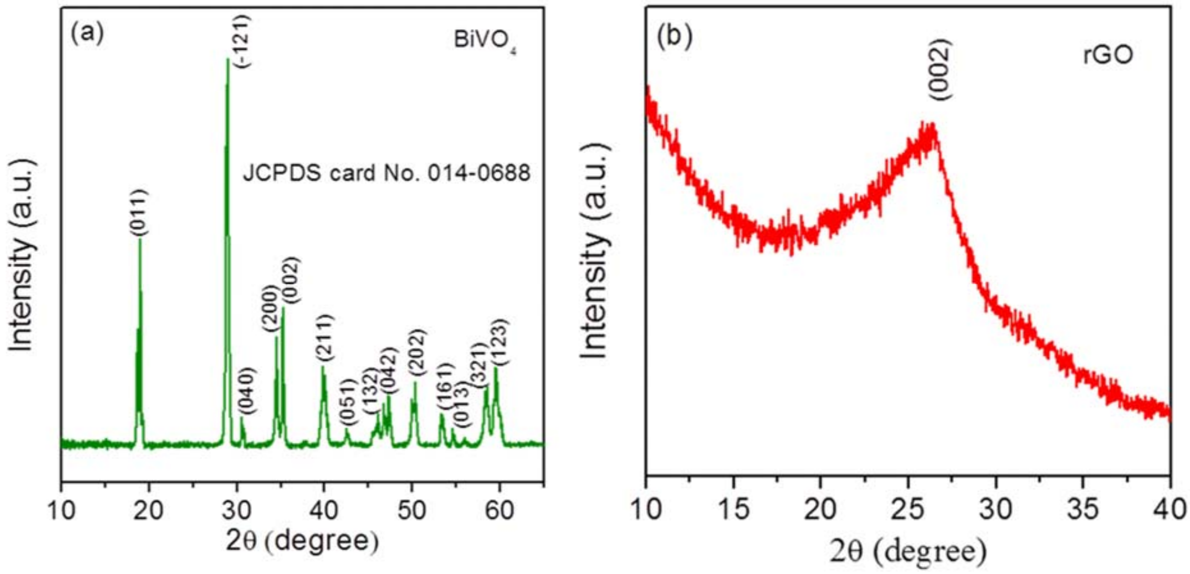

Fig. S1 XRD pattern of (a) $BiVO_4$ and (b) reduced graphene oxide (rGO)

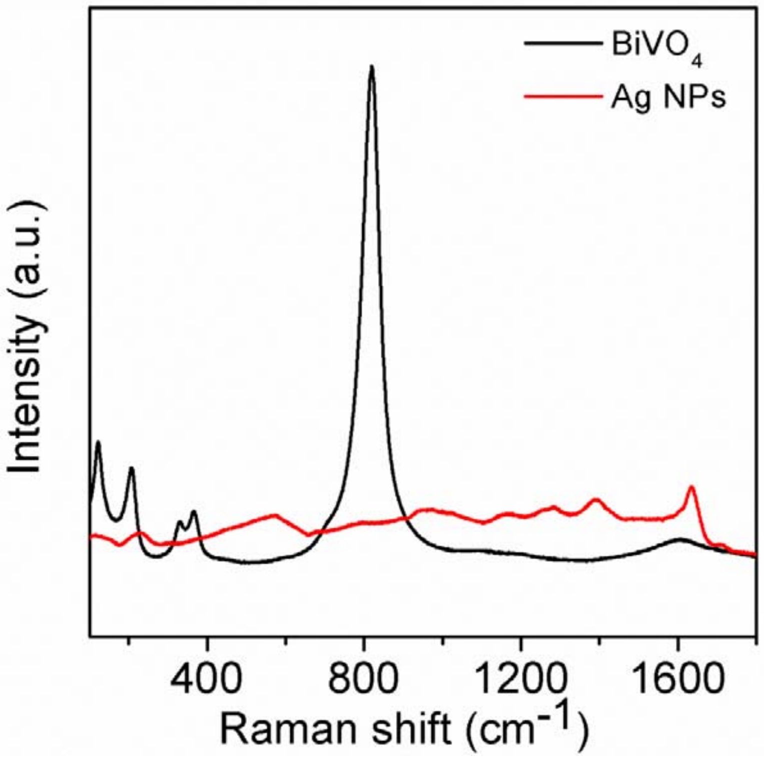

Fig. S2 Raman spectra of pure $BiVO_4$ and silver (Ag)

S2: Tauc plots and XPS analysis

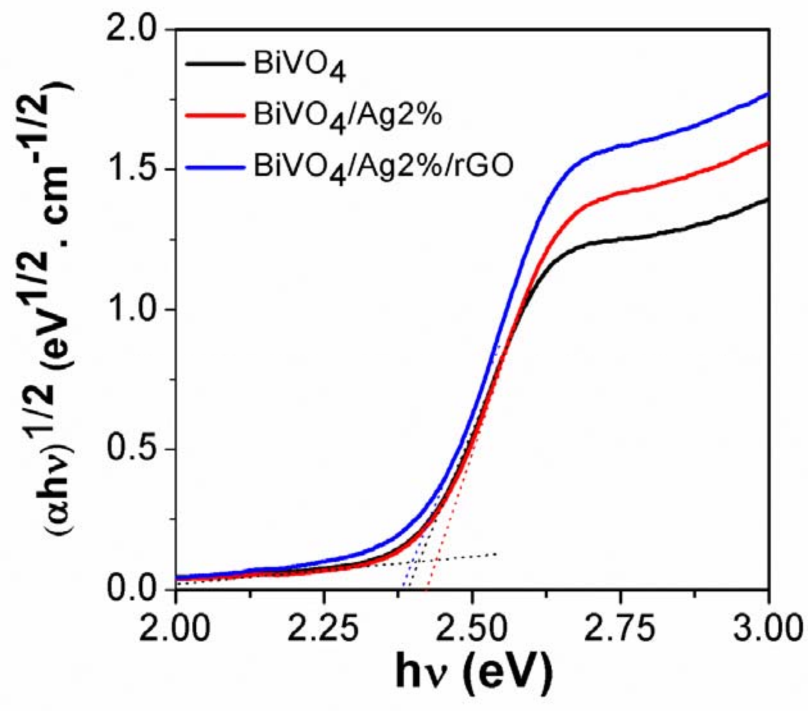

Fig. S3 Tauc plots of $BiVO_4$, $BiVO_4$/Ag2%, and $BiVO_4$Ag2%/rGO hybrid samples.

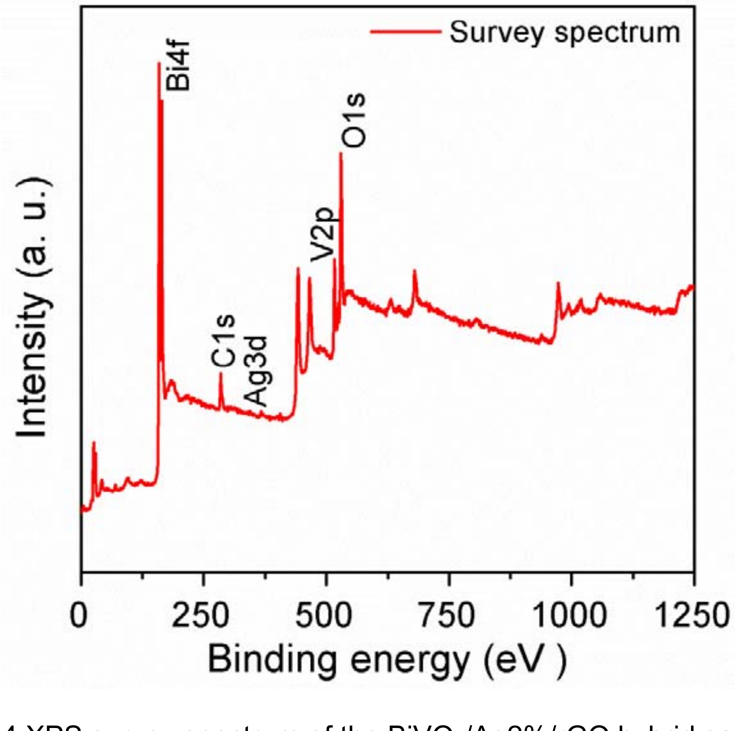

Fig. S4 XPS survey spectrum of the $BiVO_4$/Ag2%/rGO hybrid sample.

S2. Plausible reaction and growth mechanisms

The plausible reaction and growth mechanisms for the hydrothermal synthesis of $BiVO_4$/Ag/rGO architectures are given below.

$Bi(NO_3)_3.5H_2O + HNO_3 + H_2O \rightarrow BiONO_3 + HNO_3$ .... (i)

$2NH_4VO_3 + 2HNO_3 \rightarrow V_2O_5 + 2NH_4NO_3 + H_2O$

$NH_4OH \rightarrow NH_3 + H_2O$ .... (ii)

$AgNO_3 + 2NH_4OH \rightarrow Ag\ (NH_3)_2^+ + HNO_3 + H_2O$ .... (iii)

$Bi(NO_3)_3.5H_2O + 2NH_4VO_3 + AgNO_3 + rGO \rightarrow BiVO_4/Ag/rGO$ .... (iv)

First, the hydrolysis of $Bi(NO_3)_3.5H_2O$ occurs to form a soluble $BiONO_3$ species. Meanwhile, vanadate (V_2O_5) ions are added that react with bismuth, followed by the addition of NH_4OH to adjust the pH of the solution. The reaction between vanadate and bismuth occurs depending on the pH to give rise to a pale yellow precipitate constituted of tetragonal $BiVO_4$. Up to pH~3.5, no tetragonal $BiVO_4$ precipitate is formed, while above pH~4 the precipitation occurs. It is important to note that to achieve the direct hydrothermal growth of $BiVO_4$ on fluorine doped tin oxide (FTO) coated glass substrates, two factors are crucial. The first is that the FTO substrate should be placed in the hydrothermal reactor before the reaction in such a way that the FTO coated side faces in a downward direction and forms an angle of ~45° (Figure S2) with the wall of the Teflon vessel to obtain growth. The second is that it is necessary to ensure that no initial formation of precipitate occurs. If a precipitate is initially formed, no good growth and deposition of material on FTO would take place. However, during the hydrothermal treatment, after gaining sufficient thermal energy, the generated $BiVO_4$ nuclei aggregate and are converted to the highly crystalline form of monoclinic $BiVO_4$ crystals. Meanwhile, silver produces a soluble silver amine complex ($Ag(NH_3)_2^+$) upon reaction with ammonia (NH_3).[1] The formed $Ag(NH_3)_2^+$ complexed ions possess sufficient thermal energy to reach the surface of the $BiVO_4$ sites, wherein they are

reduced to Ag nanoparticles by ethanol under high temperature and pressure conditions.[1,2] It is assumed that a simultaneous nucleation of $BiVO_4$ and Ag could occur, and further growth may take place owing to the most common self-assembly and Ostwald ripening mechanisms. Similarly, in general a higher monomer (precursor) concentration in solution sustains a one dimensional 1D nanostructures growth, whereas a lower monomer concentration favors a 3D growth.[3] Herein, during the hydrothermal reaction, in the presence of a low reactant concentration and suitable reactions conditions (low temperature (150 °C), high pressure, and optimum pH) producing a high chemical potential, a fast ion movement leads to the formation of dendritic structures. Importantly, owing to the thin size and high binding capacity of rGO, it integrates well in $BiVO_4$ to develop rGO embedded architectures such as the $BiVO_4$/Ag/rGO hybrid.

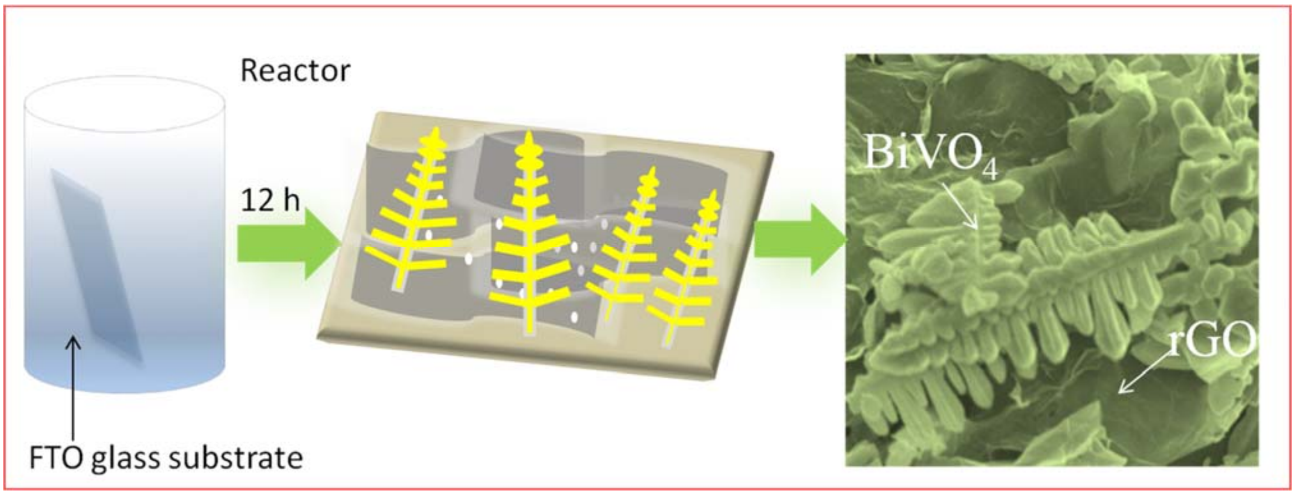

Fig. S5 Schematic representation of hydrothermal synthesis of $BiVO_4$/Ag/rGO hybrid architectures

S3: EDS spectrum of the $BiVO_4$/Ag2%/rGO hybrid architectures

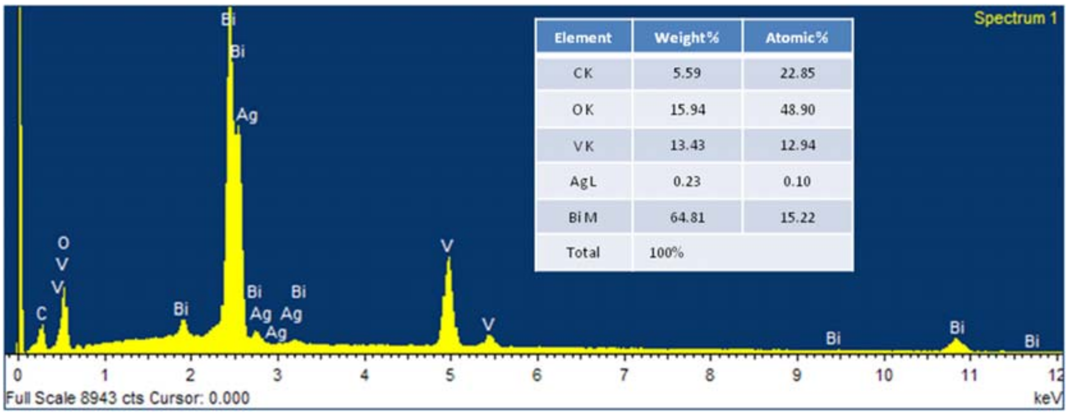

Fig. S6 EDS spectrum of the $BiVO_4$/Ag2%/rGO architectures.

S4: Photoelectrochemical water splitting (PEC-WS) characteristics

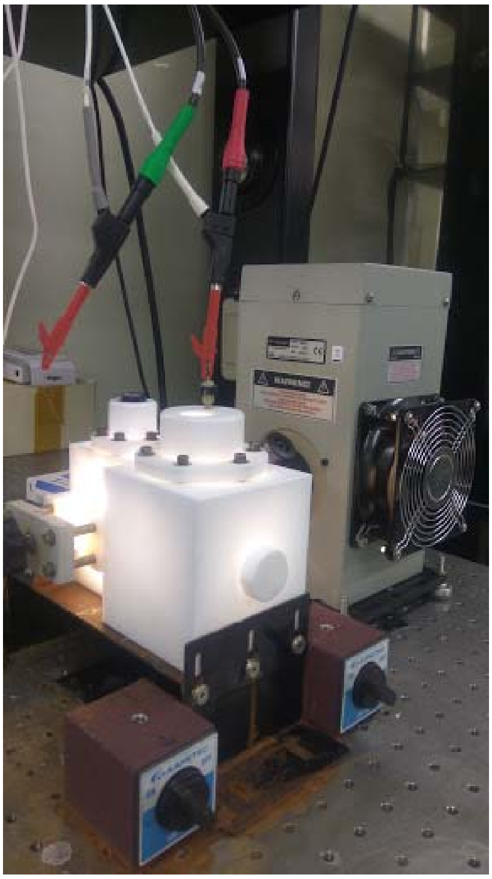

Fig. S7 Photograph of Homemade PEC-WS cell.

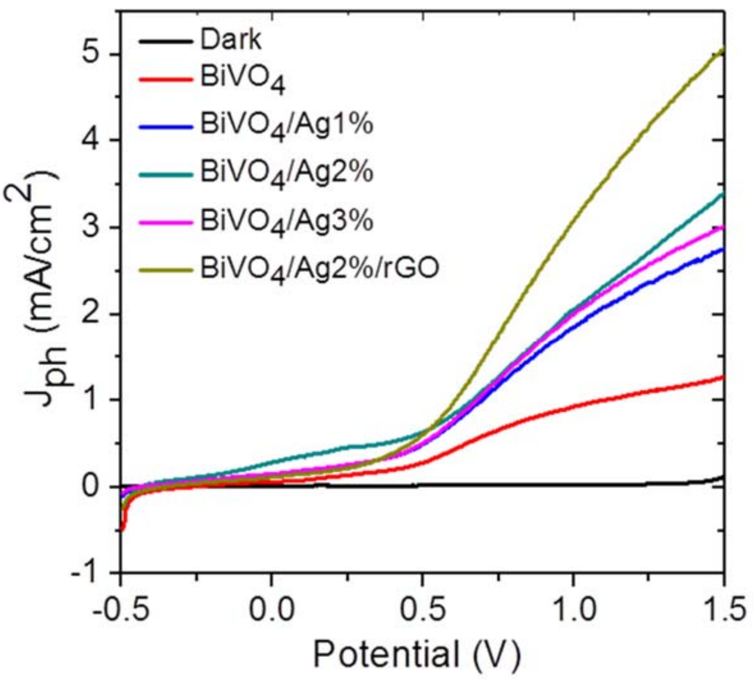

Fig. S8 Photocurrent density-voltage characteristics of the samples under dark and illumination conditions using 0.5 M Na_2SO_3 as electrolyte solution.

BET surface area study

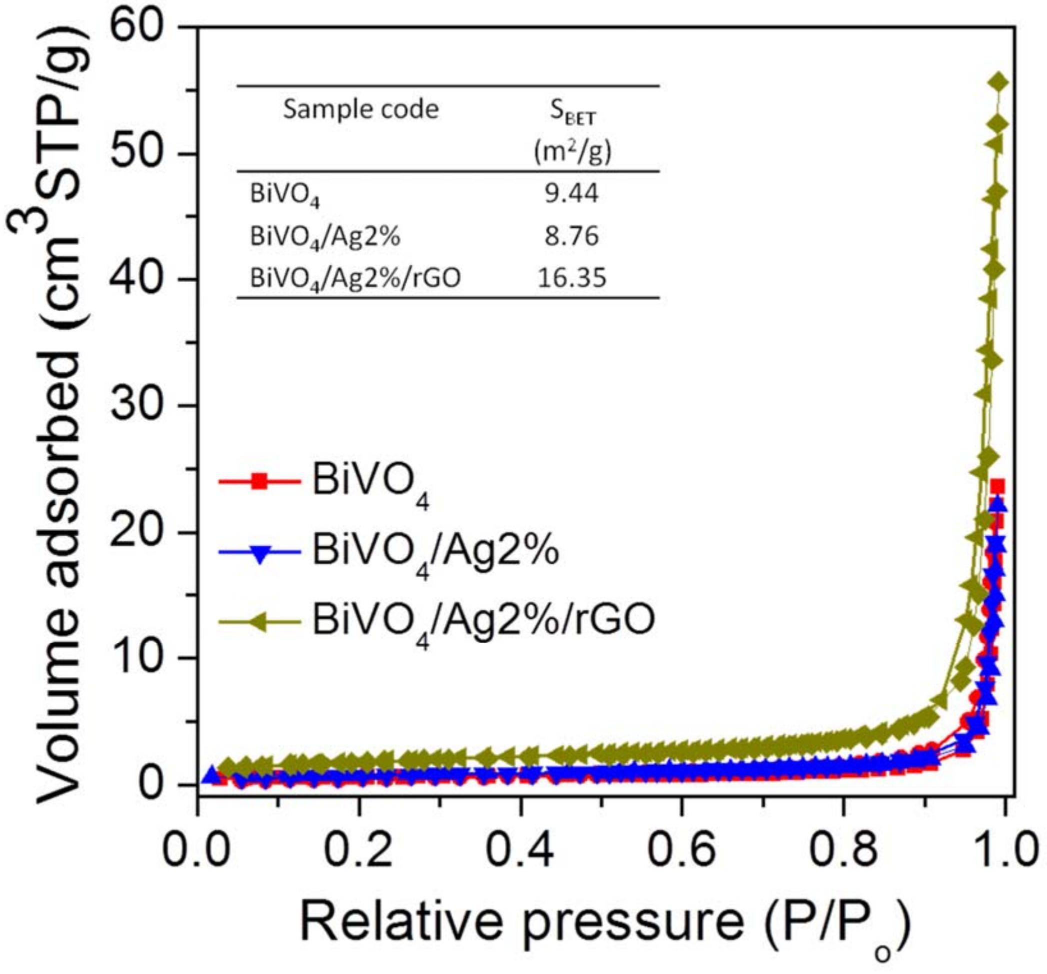

Fig.S9 Nitrogen adsorption-desorption isotherms of as prepared samples. Inset table summarizes the BET surface areas of sample

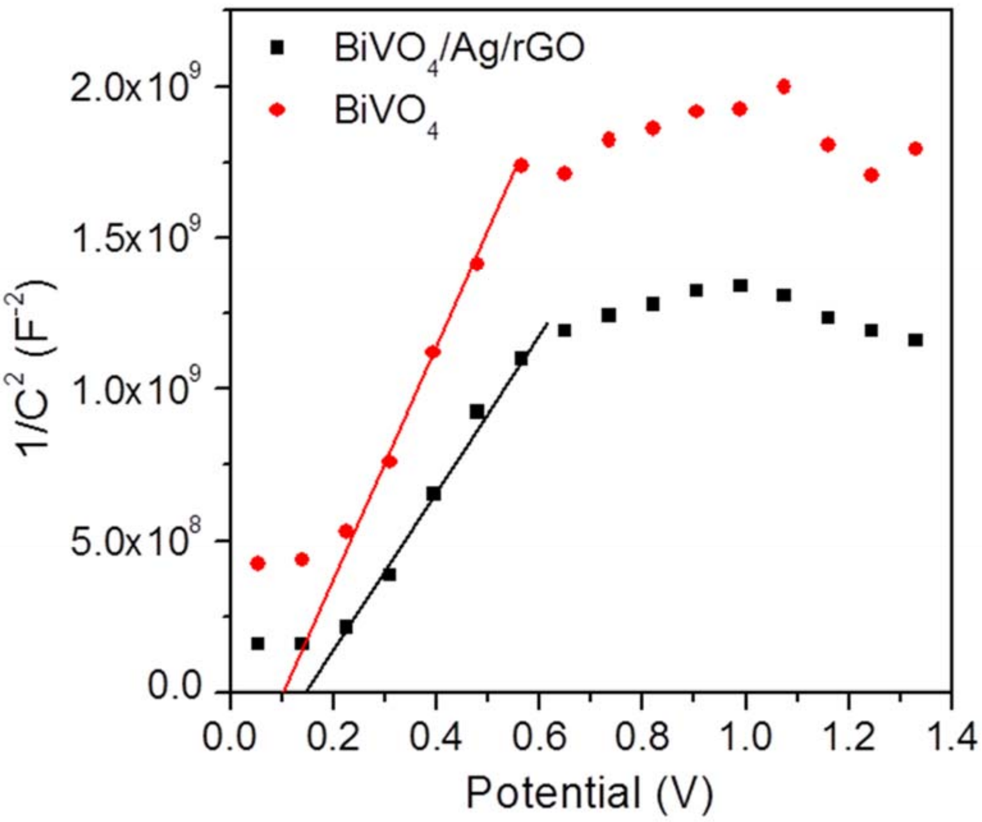

Fig. S10 Mott-Schottly plots for $BiVO_4$ and $BiVO_4$/Ag/rGO photoanode samples measured using 0.5 M Na_2SO_4 at frequency of 1000 Hz

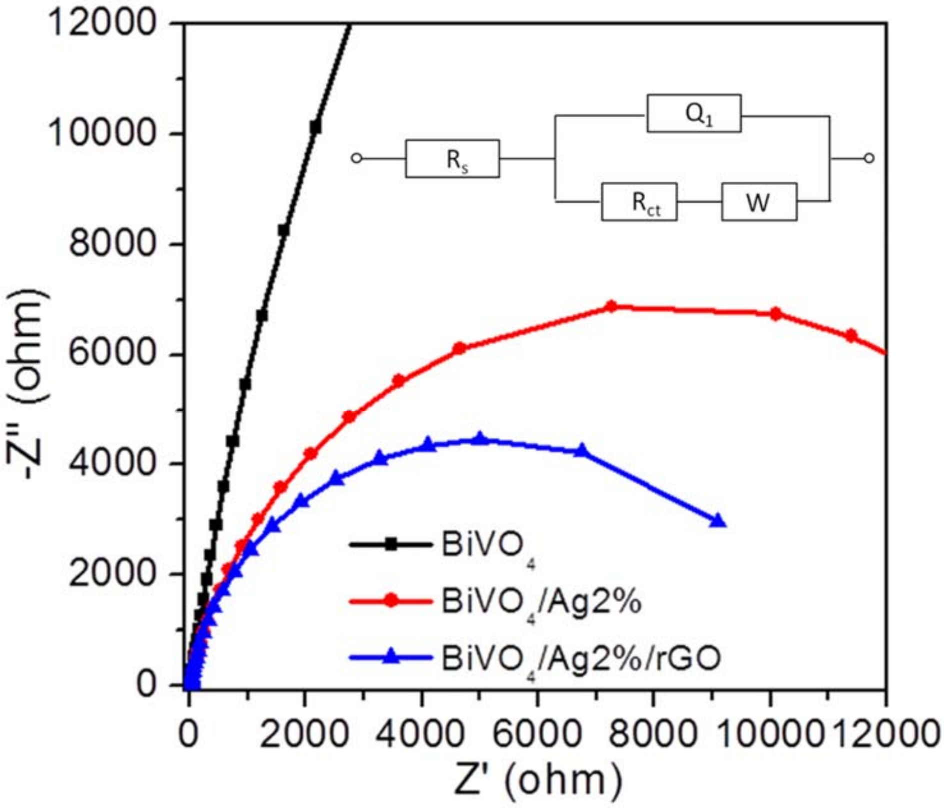

Fig. S11 Electrochemical impedance spectra of the $BiVO_4$, $BiVO_4$/Ag2%, and $BiVO_4$/Ag2%/rGO samples at 0.5 V scan using 0.5 M Na_2SO_4 as electrolyte solution. The measured data was fitted data by ZMAN^{TM} software. Inset shows the equivalent circuit.

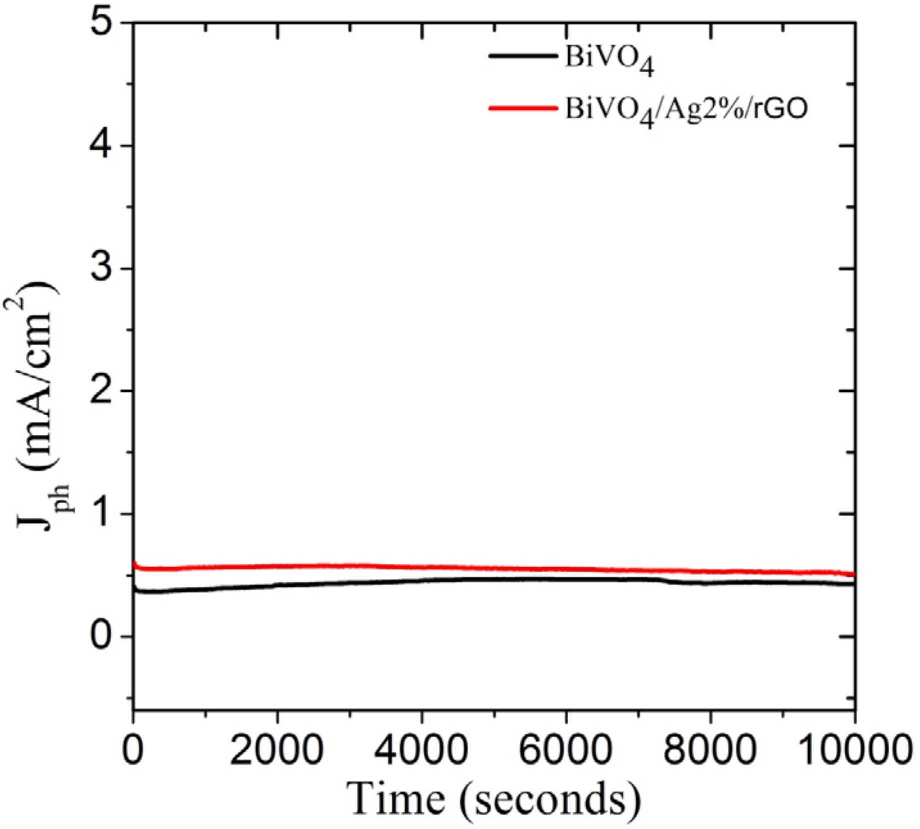

Fig. S12 Chronoamperometric (J–T) measurements using the $BiVO_4$ and $BiVO_4$/Ag2%/rGO hybrid electrode samples.

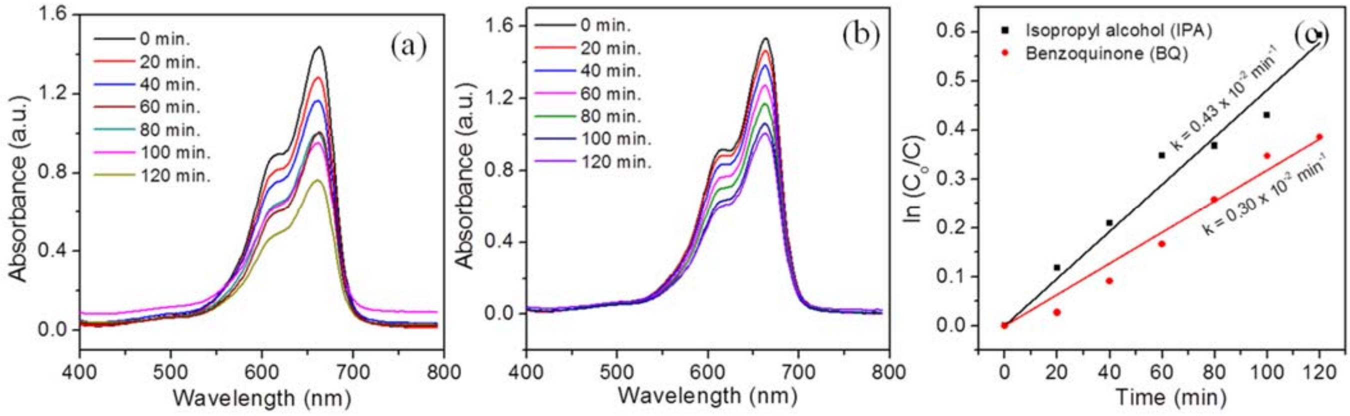

Fig. S13 Photocatalytic degradation of MB using $BiVO_4$/Ag/rGO photocatalyst in the presence of scavengers (a) isopropyl alcohol (IPA) (b) banzoquinone (BQ), (c) pseudo first order rate constants

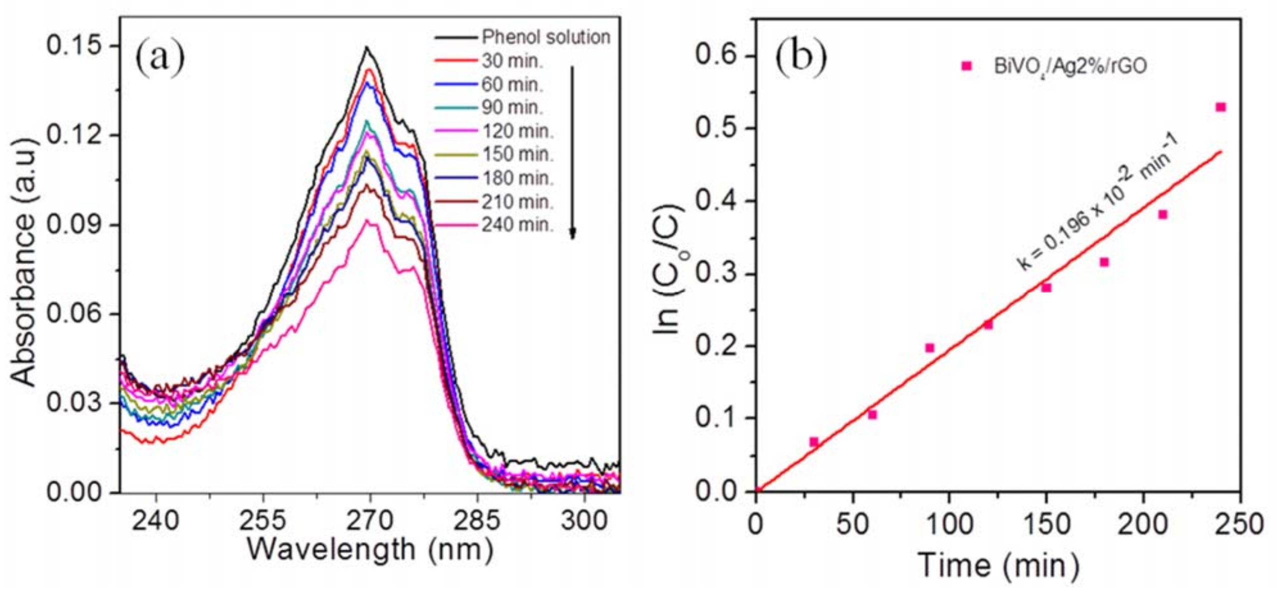

Fig. S14 (a) UV-visible absorption spectra for photocatalytic phenol degradation using BiVO4/Ag/rGO photocatalyst (b) plot of first order rate kinetics for phenol degradation

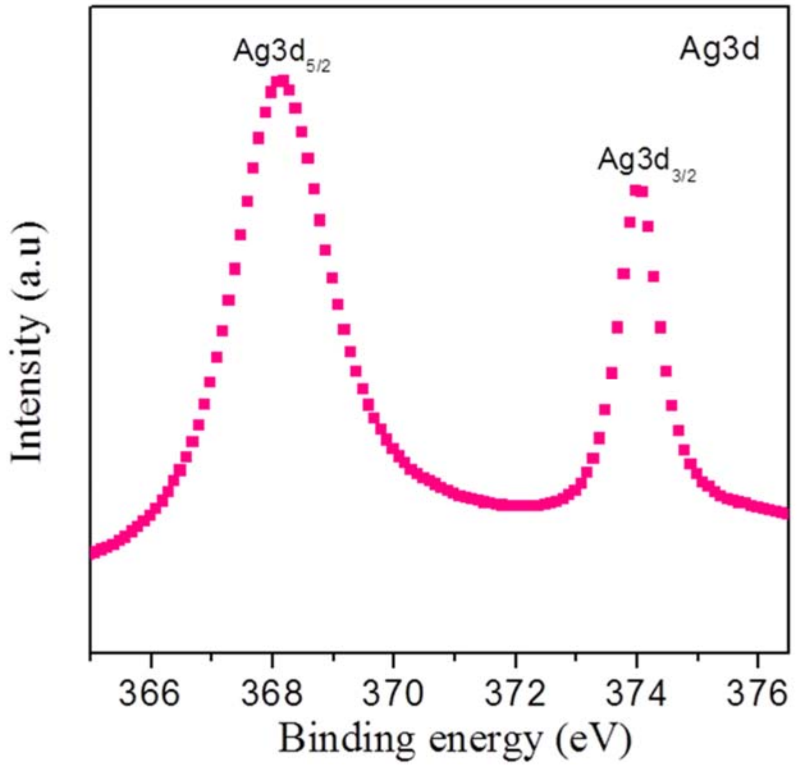

Fig. S15 XPS spectrum of $BiVO_4$/Ag/rGO photocatalyst for Ag after recycling from photocatalysis reaction

S5. Structural and morphological characterization of graphene oxide (GO)

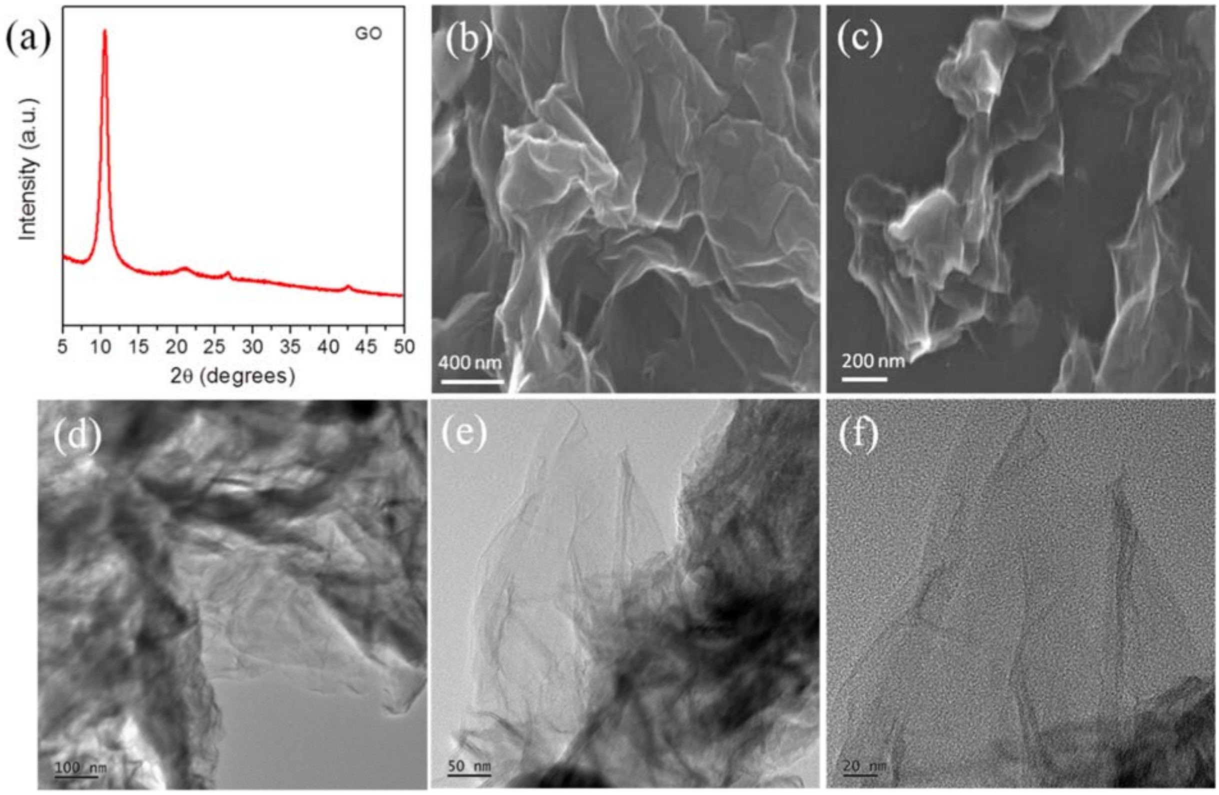

Fig. S16 (a) XRD pattern of graphene oxide (GO), (b, c) FESEM micrographs of GO, and (d-f) TEM images of GO.

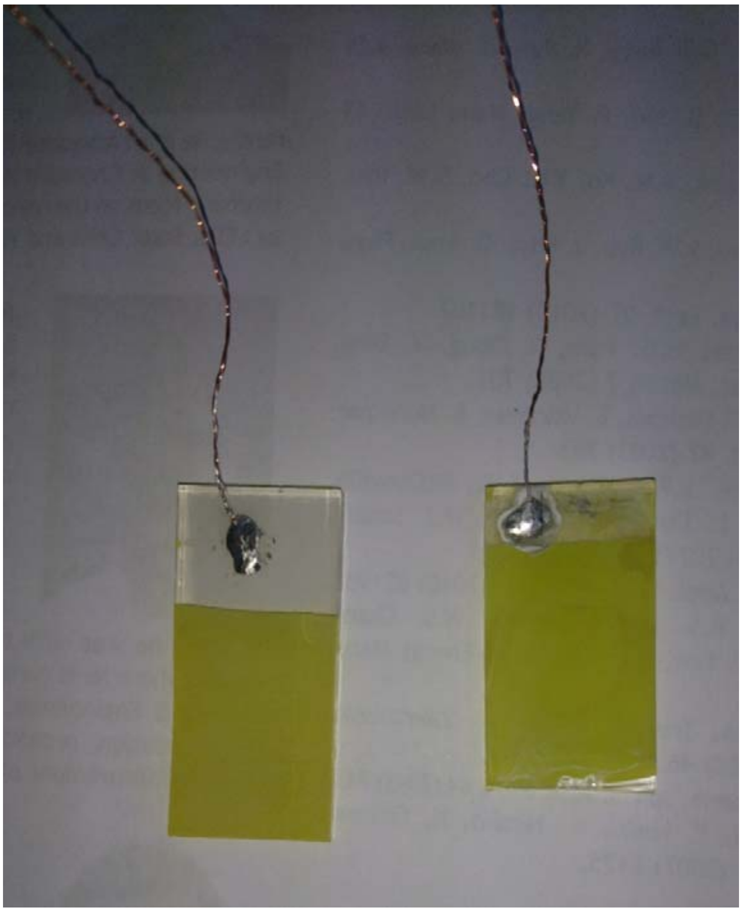

Fig. S17 The photograph of the recovered $BiVO_4$ and $BiVO_4$/Ag2%/rGO photoelectrodes after PEC-WS analysis.

Table S1: Summarized representative results in literature for PEC water splitting using $BiVO_4$ based photoelectrode systems

Photoelectrode sample	Synthesis method	Electrolyte	pH of electrolyte	Applied potential (V *vs* RHE)	Photocurrent density J_{ph} (mA/cm^2)	Reference
1D $ZnO/BiVO_4$	ZnO growth on ITO by CBD followed by $BiVO_4$ deposition using SILAR method	0.5 M Na_2SO_4	6.5	1.23 (Ag/AgCl)	1.72	[4]
$TiO_2/BiVO_4$ in combination with $CH_3NH_3PbI_3$-perovskite solar cell	Hydrothermal method for TiO_2 growth and Spray pyrolysis method for $BiVO_4$ deposition	0.1 M phosphate buffered saline (PBS)	7	1.23	1.30	[5]
$WO_3/BiVO_4$	Solvothermal deposition for WO_3 and spin coating method for $BiVO_4$ deposition	0.5 M Na_2SO_4	6.5	1	0.8	[6]
WO_3/W:$BiVO_4$		0.5 M potassium phosphate	8	1.23	3.1	[7]
$BiVO_4$/FeOOH/NiOOH	Electrochemical deposition	0.5 M phosphate Buffer<br>1 M Na_2SO_3	7	0.6	2.73<br>3.3	[8]
$BiVO_4/ZnFe_2O_4$	Electrodeposition followed by photodeposition	0.1 M KOH	13	1.23	2	[9]
W-$BiVO_4$/RGO	Drop-cast method followed by photoreduction of graphene oxide	0.5 M Na_2SO_4	6	0.9 (Ag/AgCl)	1.39	[10]
Mo:$BiVO_4$	Spin coating	1.0 M KH_2PO_4<br>0.1 M Na_2SO_3	7	1.23	1.7<br>3.1	[11]
Mo:$BiVO_4$/NiO	Electrodeposition and	1.0 M KH_2PO_4	7	0.8 (Ag/AgCl)	0.39	[12]

	calcinations					
PdOx/$BiVO_4$	Metal organic deposition (MOD)	0.1 M KPi buffer<br>0.1 M Na_2SO_3	7	1.23<br>1.23	1.15<br>1.40	[13]
Ag/$BiVO_4$	Dip-coating sol-gel technique	0.5 M Na_2SO_4	7	0 (Ag/AgCl)	0.066	[14]
$BiVO_4$/rGO	Wet chemical method combined with photocatalytic graphene oxide reduction	0.1 M Na_2SO_4		0.8 (Ag/AgCl)	0.070	[15]
$BiVO_4$/Ag/rGO	Hydrothermal method	0.5 M Na_2SO_4<br>0.5 M Na_2SO_3	6.5	1.23<br>1.23	1.45<br>4.23	Present work

Table S2 Rate constant (*K)* values for the photocatalytic MB degradation using the as-prepared photocatalyst samples.

**Sr. No.**	**Catalyst**	**Rate constant (*K)* for MB degradation [min^{-1}]**	**Std. Dev.**
1	Photolysis	0.192×10^{-2}	0.000121
2	$BiVO_4$	0.59×10^{-2}	0.000421
3	$BiVO_4$/Ag1%	0.76×10^{-2}	0.000541
4	$BiVO_4$/Ag2%	1.03×10^{-2}	0.003472
5	$BiVO_4$/Ag3%	0.86×10^{-2}	0.004715
6	$BiVO_4$/Ag2%/rGO	1.29×10^{-2}	0.000549

References:

1. Warule, S. S. Choudhari, N. S., Khare, R. T., Ambekar, J. D., Kale, B. B. More, M. A. Single step hydrothermal approach for devising hierarchical Ag-ZnO heterostructures with significant enhancement in field emission performance. *CrystEngComm* **15,** 7475–7483 (2013).

2. Patil, S. S. *et al.* Green approach for hierarchical nanostructured Ag-ZnO and their photocatalytic performance under sunlight. *Catal. Today* **260,** 126–134 (2016).

3. Patil, S. S. *et al.* Fern-like rGO/$BiVO_4$ hybrid nanostructures for high-energy symmetric supercapacitor. *ACS Appl. Mater. Interfaces* **8,** 31602–31610 (2016).

4. Yan, L., Zhao, W. & Liu, Z. 1D ZnO/$BiVO_4$ heterojunction photoanodes for efficient photoelectrochemical water splitting. *Dalt. Trans.* **45,** 11346–11352 (2016).

5. Zhang, X. *et al.* A perovskite solar cell-TiO_2@$BiVO_4$ photoelectrochemical system for direct solar water splitting. *J. Mater. Chem.* **3,** 21630–21636 (2015).

6. Su, J., Guo, L., Bao, N. & Grimes, C. A. Nanostructured WO_3/$BiVO_4$ heterojunction films for efficient photoelectrochemical water splitting. *Nano Lett.* ***11,*** 1928–1933 (2011).

7. Rao, P. M. *et al.* Simultaneously efficient light absorption and charge separation in WO_3/$BiVO_4$ core/shell nanowire photoanode for photoelectrochemical water oxidation. *Nano Lett.* **14,** 1099–1105 (2014).

8. Kim, T. W. & Choi, K.-S. Nanoporous $BiVO_4$ photoanodes with dual-Layer oxygen evolution catalysts for solar water splitting. *Science* **343,** 990–995 (2014).

9. Kim, T. W. & Choi, K. Improving stability and photoelectrochemical performance of $BiVO_4$ photoanodes in basic media by adding a $ZnFe_2O_4$ layer. *J. Phys. Chem. C* **7,** 447–451 (2016).

10. Wan, X., Niu, F., Su, J. & Guo, L. Enhanced photoelectrochemical water oxidation of bismuth vanadate via a combined strategy of W doping and surface RGO modification. *Phys. Chem. Chem. Phys.* **18,** 31803–31810 (2016).

11. Nair, V., Perkins, C. L. & Law, M. Textured nanoporous Mo:$BiVO_4$ photoanodes with high charge transport and charge transfer quantum efficiencies for oxygen evolution. *Energy Environ. Sci.* **9,** 1412–1429 (2016).

12. Xie, S., Zhai, T., Zhu, Y., Li, W. & Qiu, R. NiO decorated Mo:$BiVO_4$ photoanode with enhanced visible-light photoelectrochemical activity. *Nano Energy* **39,** 4820–4827 (2014).

13. Hyun, J. *et al.* Palladium oxide as a novel oxygen evolution catalyst on $BiVO_4$ photoanode for photoelectrochemical water splitting. *J. Catal.* **317,** 126–134 (2014).

14. Fang, L., Nan, F., Yang, Y. & Cao, D. Enhanced photoelectrochemical and photocatalytic activity in visible-light-driven Ag/$BiVO_4$ inverse opals. *Appl.Phys.Lett.* **108,** 093902–1 (2016).

15. Ng, Y. H., Iwase, A., Kudo, A. & Amal, R. Reducing graphene oxide on a visible-light $BiVO_4$ photocatalyst for an enhanced photoelectrochemical water splitting. *J. Phys. Chem. C* **1,** 2607–2612 (2010).
